# Pattern analysis approach reveals restriction enzyme cutting abnormalities and other cDNA library construction artifacts using raw EST data

**DOI:** 10.1186/1472-6750-12-16

**Published:** 2012-05-03

**Authors:** Sun Zhou, Guoli Ji, Xiaolin Liu, Pei Li, James Moler, John E Karro, Chun Liang

**Affiliations:** 1 Department of Automation, Xiamen University, Xiamen, Fujian, 361005, China; 2 Department of Botany, Oxford, OH, 45056, USA; 3 Department of Computer Science and Systems Analysis, Oxford, OH, 45056, USA; 4 Department of Microbiology, Oxford, OH, 45056, USA; 5 Department of Statistics, Miami University, Oxford, OH, 45056, USA

**Keywords:** cDNA terminus, cDNA library construction, Pattern analysis, Restriction enzyme cutting abnormality, Chimeric EST sequences

## Abstract

**Background:**

*E*xpressed *S*equence *T*ag (EST) sequences are widely used in applications such as genome annotation, gene discovery and gene expression studies. However, some of GenBank dbEST sequences have proven to be “unclean”. Identification of cDNA termini/ends and their structures in raw ESTs not only facilitates data quality control and accurate delineation of transcription ends, but also furthers our understanding of the potential sources of data abnormalities/errors present in the wet-lab procedures for cDNA library construction.

**Results:**

After analyzing a total of 309,976 raw *Pinus taeda* ESTs, we uncovered many distinct variations of cDNA termini, some of which prove to be good indicators of wet-lab artifacts, and characterized each raw EST by its cDNA terminus structure patterns. In contrast to the expected patterns, many ESTs displayed complex and/or abnormal patterns that represent potential wet-lab errors such as: a failure of one or both of the restriction enzymes to cut the plasmid vector; a failure of the restriction enzymes to cut the vector at the correct positions; the insertion of two cDNA inserts into a single vector; the insertion of multiple and/or concatenated adapters/linkers; the presence of 3′-end terminal structures in designated 5′-end sequences or vice versa; and so on. With a close examination of these artifacts, many problematic ESTs that have been deposited into public databases by conventional bioinformatics pipelines or tools could be cleaned or filtered by our methodology. We developed a software tool for Abnormality Filtering and Sequence Trimming for ESTs (AFST, http://code.google.com/p/afst/) using a pattern analysis approach. To compare AFST with other pipelines that submitted ESTs into dbEST, we reprocessed 230,783 *Pinus taeda* and 38,709 *Arachis hypogaea* GenBank ESTs. We found 7.4% of *Pinus taeda* and 29.2% of *Arachis hypogaea* GenBank ESTs are “unclean” or abnormal, all of which could be cleaned or filtered by AFST.

**Conclusions:**

cDNA terminal pattern analysis, as implemented in the AFST software tool, can be utilized to reveal wet-lab errors such as restriction enzyme cutting abnormities and chimeric EST sequences, detect various data abnormalities embedded in existing Sanger EST datasets, improve the accuracy of identifying and extracting *bona fide* cDNA inserts from raw ESTs, and therefore greatly benefit downstream EST-based applications.

## Background

ESTs are primarily cDNA sequences obtained by sequencing cDNA fragments/clones made from mRNAs. Representing transcribed portions of various genomes, ESTs are widely used for a variety of genomic researches, including novel gene discovery, gene expression studies, and genome annotation [1-8]. While cDNA/EST data generated by next-generation sequencing technologies (such as 454 or Illumina) is being deposited into the NCBI Short Read Archive (http://www.ncbi.nlm.nih.gov/Traces/sra/sra.cgi) in an unprecedented rate, the quantity of publically available EST data created by traditional Sanger sequencing is still increasing. As of November 1, 2011, there were 71,235,293 entries deposited in the GenBank dbEST, the public data repository for traditional Sanger ESTs [9]. Unfortunately, many EST datasets are poorly processed, and GenBank dbEST contains numerous errors from a range of sources. For example, double-termini adapters, the palindrome linker sequences that likely concatenate two different transcripts to form chimeric ESTs, were identified in many *Pinus teada* ESTs [10]. In another case, we were able to identify a number of spurious sequence remnants *(i.e*. vector or adapter fragments) in a large portion of the GenBank ESTs and their clusters/contigs for *Chlamydomonas reinhardtii*[11], an artifact of under-trimming during the procedures of raw EST cleanup.

In order to significantly reduce the errors in public EST databases, we proposed a protocol that processes raw EST data based on cDNA termini/ends – a set of diagnostic sequence elements that can be used to delineate cDNA insert ends and facilitate extraction of *bona fide* cDNA insert sequences from raw ESTs [11,12]. Specifically, the diagnostic sequence elements for cDNA termini include adapter/linker sequences, insert-flanking restriction enzyme recognition sites, poly (A)/(T) tails, and plasmid vector fragments immediately adjacent to cDNA inserts. Moreover, these individual elements or components must have retained their sequential order and orientation constraints and form a canonical or expected structure for a given cDNA terminus, known as the cDNA terminus structure [11]. Our previous work [11,12] focused on detecting canonical cDNA terminal structures expected from the adopted cDNA library constructional protocols and filtering out those ESTs with abnormal and complex terminal structures for downstream applications. In this study, we have collected a total of 309,976 raw *Pinus taeda* EST trace files, the majority of which have been submitted to both NCBI dbEST and Trace Archive. Using this dataset, our objective is to characterize the abnormal and complex terminus structure patterns, explore the potential underlying sources of wet-lab artifacts/errors, and develop a new EST cleaning software tool based on pattern analysis approach. Using our new tool, we have reprocessed 230,783 *Pinus taeda* and 38,709 *Arachis hypogaea* GenBank ESTs, and detected a significant number of problematic EST sequences. Clearly, characterization of abnormal and complex terminal structures will improve current EST cleaning steps and facilitate the quality control of error-prone ESTs.

## Results and Discussion

### Pattern analysis of abnormal cDNA terminal structures

**Figure 1:**
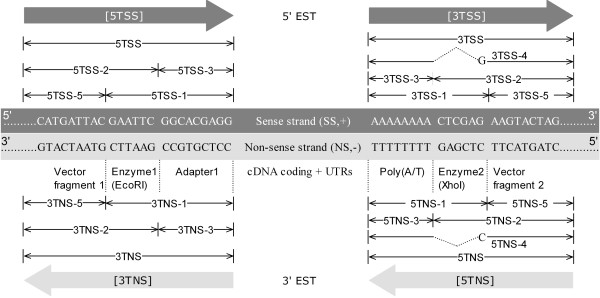
**The expanded definitions of cDNA terminal structures.** The original four canonical cDNA termini – 5TSS, 3TSS, 5TNS and 3TNS [12] have been expanded by adding some sub-categories.

In our previous studies [11,12], we defined four canonical cDNA termini: **5**′ **t**erminus of the cDNA in the **s**ense **s**trand (**5TSS**), **3**′ **t**erminus of the cDNA in the **s**ense **s**trand (**3TSS**), **5**′ **t**erminus of the cDNA in the **n**on-sense (anti-sense) **s**trand (**5TNS**), and **3**′ **t**erminus of the cDNA in the **n**on-sense **s**trand (**3TNS**). In particular, **5TSS** and **3TSS** denote the 5′ and 3′ ends of the relevant mRNA, respectively, in the sense strand, whereas **5TNS** and **3TNS** delineate the 3′ and 5′ ends of an mRNA, respectively, and whose sequences are read in the 5′ → 3′ direction in the non-sense strand. In order to better characterize the abnormal and complex terminus structures, in this study we have expanded our cDNA terminus definitions by adding more sub-components, as shown in Figure 1. For example, **3TSS-1** represents the combination of a poly(A) tail and a *Xho*I site (CTCGAG, *Enzyme2*); **3TSS-2** denotes the combination of a *Xho*I site (CTCGAG, *Enzyme2*) and the adjacent plasmid vector fragment marked as Vector fragment 2 (*VF2*); **3TSS-3** represents the poly(A) tail; **3TSS-4** denotes direct adjunction of a poly(A) tail, a guanine (G) instead of a *Xho*I site (CTCGAG, *Enzyme2*), and the vector fragment *VF2*, which is impossible in theory; and **3TSS-5** stands only for the vector fragment *VF2*. In Figure 1*VF1* and *VF2* are referred to the left and right vector borders of the cloning sites.

Using the same or similar cDNA library construction protocol illustrated in Figure 1, 309,976 raw ESTs for *Pinus teada* were generated by three different labs – UGALAB (172,229), NCSUFBG (75,001) and TIGR_JCVIJTC (62,746). Among the UGALAB ESTs, we found that 82% (141,914 out of 172,229) contain detectable cDNA termini. Of those, about 38% (54,112 out of 141,914) match the expected terminal structures described in Figure 1, while 62% (87,802 out of 141,914) possessed abnormal terminal structures that were different from the expected structures. In contrast, among the ESTs from NCSUFBG and TIGR_JCVIJTC, 94% (70,589 out of 75,001) and 99% (62,253 out of 62,746) have detectable cDNA termini respectively. Of the identified ESTs, NCSUFBG has 68% (47,845 out of 70,589) with the expected cDNA terminal structures and 32% (22,744 out of 70,589) with abnormal terminal structures, whereas TIGR_JCVIJTC has 44% (27,368 out of 62,253) with expected cDNA terminal structures and 56% (34,885 out of 62,253) with abnormal terminal structures. Clearly, ESTs from UGALGB display more complex cDNA terminal patterns than the other two data sources.

Table 1 lists the most frequent abnormal patterns of cDNA termini detected in the 172,229 UGALAB ESTs generated using the exact protocol shown in Figure 1. The most frequent pattern (Case No.1, making up 30.74% of all 5′-end ESTs) is a single or multiple poly(A) fragments identified near the end of 5′-EST sequences *without* either a flanking *Xho*I enzyme site or the vector fragment *VF2* in proximity (Additional file 1: Figure S1 A). The second most frequent pattern (Case No. 2, 9.28%) is the replacement of a *Xho*I site (CTCGAG, *Enzyme2*) by a single Cytosine (C) that is flanked by the vector fragment *VF2* in the front and by the poly(T) tail at the end in 3′-end ESTs (see **5TNS-4** in Figure 1 and Additional file 1: Figure S1 B). The counterpart pattern of Case No. 2 in 5′-end ESTs is Case No. 8 (0.90%) where a *XhoI* site (CTCGAG) is replaced by a single Guanine (G) that is flanked by the poly(A) tail in the front and by the vector fragment *VF2* at the end in the sense strand of cDNA (see **3TSS-4** in Figure 1 and Additional file 1: Figure S1 C). The cDNA terminal pattern Cases No. 3 (8.21%, Additional file 1: Figure S1 D), No. 4 (1.59%, Additional file 1: Figure S1 E), No. 6 (1.49%, Additional file 1: Figure S1 F) and No. 7 (0.98%, Additional file 1: Figure S1 G) reflect the common theme: cDNA inserts are not flanked by a poly(A)/poly(T) tail near 3TSS or 5TNS, but flanked by either the vector fragment *VF2* or by an *Xho*I site plus *VF2*. This theme is theoretically impossible because polyadenylated mRNAs were captured for reverse transcription reaction during the cDNA library construction in terms of the protocol shown in Figure 1. Among the most frequent patterns, Case No. 5 (1.55%, Additional file 1: Figure S1 H) indicates that designated 5′-end ESTs possess 3′-end like cDNA terminal structures. While the frequency distributions listed here are based on the UGALAB ESTs, an analysis of ESTs from NCSUFBG or TIGR_JCVIJTC leads to similar results.

**Table 1:** **The top patterns of abnormal sequences detected in 172, 229*Pinus taeda*ESTs generated from UGALGB**

**Case No.**	**EST Direction**	**Terminal Pattern**^1^	**Number**^2^	**%**^3^	**Description**	**Example**^4^
1	5'	N,3TSS-3,N,…,3TSS-3,N	27426	30.74%	Single or multiple poly(A) fragment(s).	Additional file 1: Figure S1 A
2	3'	(N,)5TNS-4,N	7707	9.28%	*Xho*I site (CTCGAG) is replaced by C.	Additional file 1: Figure S1 B
3	3'	(N,)5TNS-5,N	6814	8.21%	The vector fragment 2 (*VF2)* has been identified, without adjacent poly(T) tail and *Xho*I site detected.	Additional file 1: Figure S1 D
4	5'	N,3TSS-5,V	1414	1.59%	The vector fragment 2 (*VF2*) has been identified, without poly(A) tail and *Xho*I detected.	Additional file 1: Figure S1 E
5	5'	N,5TNS-1,N	1386	1.55%	3′-like terminus, i.e., poly(T) and *Xho*I site, without the vector fragment 2 (*VF2*) detected.	Additional file 1: Figure S1 H
6	3'	5TNS-2,N	1238	1.49%	*Xho*I site and the vector fragment 2 (VF2) identified, without a poly(T) tail detected.	Additional file 1: Figure S1 F
7	5′	N,3TSS-5,N	873	0.98%	The vector fragment 2 (*VF2*) has been identified, without adjacent poly(A) tail and *Xho*I site detected	Additional file 1: Figure S1 G
8	5'	N,3TSS-4,V	800	0.90%	*Xho*I site (CTCGAG) is replaced by G.	Additional file 1: Figure S1 C

^1^*V* stands for vector sequence while *N* stands for non-vector sequence.

^2^ Total sequence numbers for a given case

^3^ Of 172,229 ESTs, 83,021 are designated as 3′-ESTs (with ".b" in their sequence names) whereas 89,208 as 5′-ESTs ("*.g*" in their sequence names). The percentage is calculated using the total sequence number for each case divided by all 3′-end or 5′-end ESTs.

^4^All examples are displayed in Additional file 1: Figure S1.

### Restriction Enzyme Cutting Abnormality (RECA)

**Figure 2:**
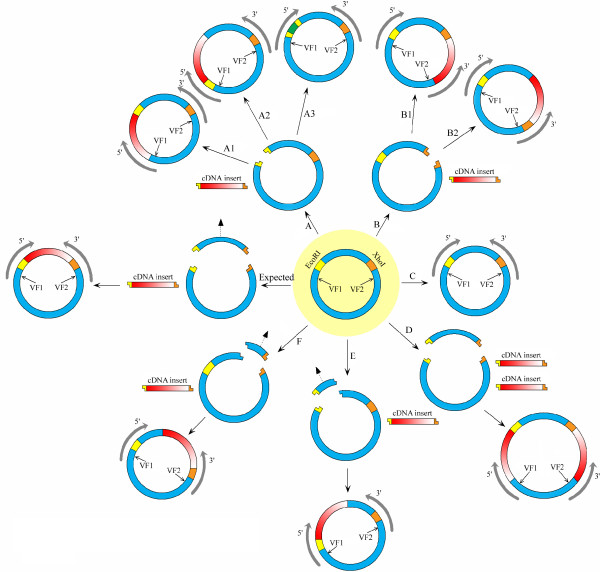
**The expected construction of cDNA insertion and all types of Restriction Enzyme Cutting Abnormality (RECA).** The label “Expected” means the expected construction of cDNA library. Sequencing direction is indicated as 3′ or 5′ with an arrow. VF1 (Vector fragment 1) and VF2 (Vector fragment 2) are referred to the left and right vector borders of the cloning sites. A, B, C, D, E and F are special types of RECA, defined as following: **RECA-Type A**: *EcoR*I site is cut off but *Xho*I site remains intact. **A1**: cDNA is inserted with inversion; **A2**: cDNA is inserted without inversion; **A3**: Adapter/linker fragments are inserted. **RECA-Type B**: *Xho*I site is cut off but *EcoR*I site remains intact. **B1**: cDNA is inserted with inversion; **B2**: cDNA is inserted without inversion. **RECA-Type C**: Neither of the two enzyme sites is cut off. **RECA-Type D**: Both the two enzyme sites are cut off, but the excised vector fragment remains. **RECA-Type E**: *Xho*I cuts off the vector at wrong site. **RECA-Type F**: *EcoR*I cuts off the vector at wrong site. The yellow color indicates *EcoR*I recognition site or *EcoR*I sticky end. The brown color stands for *Xho*I recognition site or *Xho*I sticky end. The blue represents the plasmid vector. Dark green denotes for adapter/linker fragment. cDNA insert direction is represented by red color with gradual changes: cDNA sense strand is from deep red to light red whereas cDNA non-sense strand is from light red to deep red.

Because of low frequencies in occurrence, a number of more complicated abnormal patterns of cDNA termini are not listed in Table 1. Among them, interestingly, is a set of patterns that reveal *R*estriction *E*nzyme *C*utting *A*bnormality (**RECA**), as summarized in Table 2 and shown in Figure 2. It is known that, for a given cDNA library construction protocol using a specific plasmid vector (see Figure 1 for an example), the vector sequence between the two restriction enzyme (*e.g.*, *EcoR*I and *Xho*I) recognition sites should be completely removed prior to the concatenation of a cDNA insert. However, our pattern analysis approach revealed that many variants of cDNA terminus structure patterns can indicate possible wet-lab abnormalities during the restriction enzyme digestion procedure.

**Table 2:** Summary of restriction enzyme cutting abnormality (RECA)

**Type**	**Feature**	**Pattern**	**cDNA contained in the sequence**	**Example (Additional file 1: Figure S2)**
**A**	**A1:** cDNA is inversely inserted	5′: N,3TNS-1,V,3TSS-2 3′: 5TNS-2,V,5TSS-1,N	5′: a cDNA ***non-sense*** strand 3′: a cDNA ***sense*** strand	FLD1_38_A06.g1_A029 (Fig. S2 A) RTDR3_19_H01.b1_A022 (Fig. S2 B)
	**A2:** cDNA is inserted without inversion	5′: 5TSS,N,V,3TSS-2 3′: 5TNS-2,V,N,3TNS	5′: a cDNA sense strand 3′: a cDNA non-sense strand	RTDR1_20_F07.g1_A015 (Fig. S2 C) RTMNUT1_27_H12.g1_A029 (Fig. S2 D) STRS1_37_H01.b1_A034 (Fig. S2 E)
	**A3:** Adapter/linker fragments are inserted	5′:5TSS,3TNS-1,V,3TSS-2 3′:5TNS-2,V,5TSS-1,3TNS	No cDNA	NXRV076_A06_F (Fig. S2 F)
**B**	**B1:** cDNA is inversely inserted	5′: 5TSS-2,V,5TNS-1,N 3′: N,3TSS-1,V,3TNS-2	5′: a cDNA ***non-sense*** strand 3′: a cDNA ***sense*** strand	NXRV_013_E07_F (Fig. S2 G) RTDS1_2_A09.b1_A015 (Fig. S2 H)
	**B2:** cDNA is inserted (non-inversely)	5′: 5TSS-2,V,N,3TSS 3′: 5TNS,N,V,3TNS-2	5′: a cDNA sense strand 3′: a cDNA non-sense strand	RTFEPL1_26_F12.g1_A029 (Fig. S2 I) RTFEPL1_26_F12.b1_A029 (Fig. S2 J)
**C**	Neither of the two enzyme sites is cut off.	5′: 5TSS-2,V,3TSS-2 3′: 5TNS-2,V,3TNS-2	No cDNA	NXCI_011_D03_F (Fig. S2 K) NXCI_029_D07_F (Fig. S2 L)
**D**	Both the two enzyme sites are cut off, but the vector fragment that should be removed still remains.	5′: N,3TNS-1,V,5TNS-1,N 3′: N,3TSS-1,V,5TSS-1,N	5′: ***two*** cDNA ***non-sense*** strands 3′: ***two*** cDNA ***sense*** strands	RTCNT1_24_B05.g1_A029 (Fig. S2 M)
**E**	XhoI cuts off at wrong site from the vector.	5′: N,V,3TSS-2 3′: 5TNS-2,V,N	5′: a cDNA sense strand 3′: a cDNA non-sense strand	COLD1_26_G12.b1_A029 (Fig. S2 N)
**F**	EcoRI cuts off at wrong site from the vector.	5′: V,N 3′: N,V	5′: a cDNA sense strand 3′: a cDNA non-sense strand	RTCA1_14_E09.g1_A029 (Fig. S2 O)

#### RECA-Type A: *EcoR*I site is cut off but *Xho*I site remains intact

**Figure 3:**
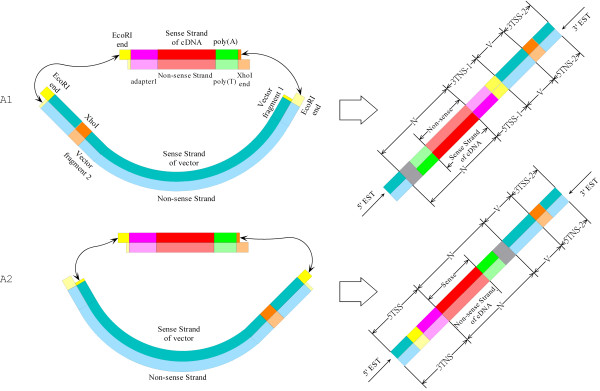
**Detailed illustration of two sub-categories of Type A Restriction Enzyme Cutting Abnormality (RECA-Type A).** RECA-Type A indicates that *EcoR*I site of the vector is cut off whereas *Xho*I site is kept. **A1** is the special case where cDNA is inserted with inversion while cDNA is inserted without inversion for **A2**. Because *Xho*I and *EcoR*I sticky ends cannot be smoothly ligated, so a random sequence fragment between the vector and cDNA end have been detected. Blue stands for the plasmid vector, yellow for *EcoR*I, brown for *Xho*I, red for cDNA, gray for a random sequence fragment, pink for *Adapter1*, and green either for poly(A) in sense strand of cDNA or for poly(T) in non-sense strand of cDNA.

RECA-Type A is the case where the *Eco*RI restriction enzyme site is cut successfully by an endonuclease (restriction enzyme) while the *Xho*I site is kept intact. As a result, the vector fragment between the two recognition sites incorrectly remain in the vector, and the cDNA inserts can be inserted into the vector by ligating to the two sticky ends of the *EcoR*I cut-off site. Depending on how a cDNA insert is ligated to the resultant sticky ends, we can identify A1 and A2 sub-categories, as shown in Figure 3. In addition, we found an additional special case in which the adapter/linker fragments replace the cDNA insert in the ligation. This case is categorized as A3 sub-category and shown in Figure 2.

##### **A1:** The double-stranded cDNA insert is inserted with inversion

As shown in Figure 3, the double-strand cDNA insert is concatenated by the sticky ends of the vector in such a way that the sense strand (*i.e.*, the one containing a poly(A) tail) and non-sense strand (*i.e.*, the one containing a poly(T) tail) of the cDNA are ligated to non-sense and sense strand of the double-strand plasmid vector respectively. Consequently, the 5′-end sequence contains cDNA sequence in the non-sense strand with a cDNA terminal pattern like *N + 3TNS-1 + V + 3TSS-2*, whereas the 3′-end sequence possesses cDNA sequence in the sense strand with a cDNA terminal pattern like *5TNS-2 + V + 5TSS-1 + N*. Here, *V* stands for vector fragment sequence and *N* for non-vector sequence. As shown in Additional file 1: Figure S2 A and B, the 5′-end sequence *FLD1_38_A06.g1_A029* and 3′-end *RTDR3_19_H01.b1_A022* exemplify this case. The 5′-end EST *FLD1_38_A06.g1_A029* actually contains a 3′-end like cDNA sequence in the non-sense strand with a detected pattern of *N + 3TNS-1 + V + 3TSS-2*, whereas 3′-end EST *RTDR3_19_H01.b1_A022* possesses a 5′-end like cDNA sequence in the sense strand with a detected pattern of *5TNS-2 + V + 5TSS-1 + N***.** In the GenBank submission, *FLD1_38_A06.g1*_*A029* has not been trimmed of its 3TNS-1 component (*i.e.* CCTCGTGCC - *Adapter1* and GAATTC - *EcoR*I site) at the end (http://www.ncbi.nlm.nih.gov/nucest/48933478). More importantly, it has been incorrectly designated as a 5′-end sequence while it actually represents a 3′-end sequence.

##### **A2:** The double-stranded cDNA insert is inserted without inversion

In contrast to the aforementioned A1 sub-category, A2 represents the case where the sense and non-sense strands of the cDNA are ligated to the sense and non-sense strands of the plasmid vector at the *EcoR*I cut-off site – an insertion without inversion. Consequently, the resultant cDNA terminal pattern of 5′-end ESTs is *5TSS + N + V + 3TSS-2* (*e.g. RTDR1_20_F07.g1_A015* and *RTMNUT1_27_H12.g1_A029* in Additional file 1: Figure S2 C and D), whereas it is *5TNS-2 + V + N + 3TNS* for 3′-end ESTs (*e.g. STRS1_37_H01.b1_A034* in Additional file 1: Figure S2 E). As shown in Figure 3, after the vector is cut off at *EcoR*I restriction enzyme site, there are two resultant *EcoR*I sticky ends available in two ends of the plasmid vector (*i.e.* the yellow parts in the beginning and at the end of the vector). One of these sticky ends can ligate to the counterpart *EcoR*I sticky end of the cDNA insert (*i.e.* the yellow part in the front of the cDNA insert), and the other one should be available to ligate to the *Xho*I sticky end of the cDNA insert (*i.e.* the brown part at the end of the cDNA insert). However, the *EcoR*I and *Xho*I sticky ends cannot be ligated naturally and smoothly together. Owing to this incompatibility, interestingly, we have found that some uncertain random sequences, denoted by gray color in Figure 3, have been generated during the ligation between the *EcoR*I and *Xho*I sticky ends.

##### **A3:** adapter/linker fragments are inserted

Instead of a cDNA insert, adapter/linker fragments are found in some sequences to be ligated at the *EcoR*I cut-off site to the vector. In the example shown in Additional file 1: Figure S2 F, the 5′-end sequence *NXRV076_A06_F* displays the cDNA terminal pattern of *5TSS + 3TNS-1 + V + 3TSS-2,* and there is no cDNA insert detectable around *EcoR*I cut-off site. Unfortunately, in the GenBank submission (http://www.ncbi.nlm.nih.gov/nucest/21689178), the region between 157 and 432 was taken as the final clean sequence, which was identified as a vector fragment sequence using our method.

#### RECA-Type B: the *Xho*I site is cut off while the *EcoR*I site remains intact

In Figure 2, similar to the RECA-Type A, there are two sub-categories whose definitions are based on whether or not the cDNA insert is inversely ligated at the *Xho*I cut-off site: **B1** is for the insertion (ligation) with inversion (see *NXRV_013_E07_F* and *RTDS1_2_A09.b1_A015* in Additional file 1: Figure S2 G and H for examples) and **B2** for the insertion (ligation) without inversion (see *RTFEPL1_26_F12.g1_A029*, and *RTFEPL1_26_F12.b1_A029* in Additional file 1: Figure S2 I and J for examples).

#### RECA-Type C: Neither of the enzyme sites is cut off

As shown in Figure 2, sometimes neither of the two restriction enzymes is successfully cut from the vector and consequently no cDNA fragment is inserted. This case is exemplified by *NXCI_011_D03_F* and *NXCI_029_D07_F* in Additional file 1: Figure S2 K and L, both of which have a terminal pattern of *5TSS-2 + V + 3TSS-2*.

#### RECA-Type D: Both *EcoR*I and *Xho*I sites are cut off, but the vector fragment that theoretically should be removed still remains

This type is a combination of RECA-Type A1 and B1. The abnormality appears to be caused by the fact that, after the cutoff at both *EcoR*I and *Xho*I sites, two cDNA inserts are inserted or ligated at both cutoff sites separately. Consequently, no vector fragment is actually cut off and removed (see Figure 2 D). Depending on the orientation of the two cDNA inserts, there are a number of complex sub-categories. Currently, one relevant terminal pattern that has been detected is *N + 3TNS-1 + V + 5TNS-1 + N*, exemplified by 5′-end sequence *RTCNT1_24_B05.g1_A029* in Additional file 1: Figure S2 M.

#### RECA-Type E: The restriction enzyme *Xho*I did not cut at its recognition site

The vector sequence fragment between the restriction enzyme recognition sites of *EcoR*I and *Xho*I should theoretically be removed from the vector, but our analysis shows that part of this vector fragment flanking *Xho*I recognition site still remains in some ESTs. One reasonable explanation of this phenomenon is that the restriction enzyme *Xho*I failed to cut off the vector at its recognition site (see Figure 2, Type E). As shown in Additional file 1: Figure S2 N, *COLD1_26_G12.b1_A029* appears to represent this case, in which a 3′-EST displays the cDNA terminal pattern as *5TNS-2 + V + N.*

#### RECA-Type F: The restriction enzyme *EcoR*I did not cut at its recognition site

Similar to RECA-Type E, when the restriction enzyme fails to cut the vector at the *EcoR*I site, part of the vector fragment flanking *EcoR*I site can be retained in some ESTs (see Figure 2, Type F). As shown in Additional file 1: Figure S2 O, *RTCA1_14_E09.g1_A029* supports this case, showing that a 5′-EST can have a cDNA terminal pattern like *V + N* + *3TSS*. **3TSS** is not necessarily detectable due to either low quality sequence region or longer cDNA insert.

Overall, we detected 1,087 EST sequences with RECA cases. Summing up all sequence numbers of each RECA types in Table 3, we can see that RECA-Type A (52.8%), RECA-Type B (21.8%) and RECA-Type C (23.0%) are the most common types. RECA-Type D, E and F account for about 1.7% of all cases, whereas all other unclassified, complicated cases make up about 0.6%. Of all the three labs, UGALGB has 765 RECA sequences and NCSUFBG has 322, whereas no RECA case is detected in the ESTs from TIGR_JCVIJTC. Interestingly, different labs have different RECA types. For example, RECA-Type A2, D, E and F are found in UGALAB ESTs but not in NCSUFBG ESTs whereas RECA-Type A3 and C are identified in NCSUFBG ESTs but not in UGALAB ESTs.

**Table 3:** Numbers of each type of RECA sequences

	**RECA Type**										**Total Sequence**
	**A1**	**A2**	**A3**	**B1**	**B2**	**C**	**D**	**E**	**F**	**Other^1^**	
UGALAB	432	101	0	107	100	0	2	9	9	5	765
NCSUFBG	3	0	37	18	12	250	0	0	0	2	322
Total	436	101	37	125	112	250	1	9	9	7	1087

^1^ Other types include two cases: (1) sequences with complicated patterns whose type is hard to be determined; (2) sequences with too bad quality to determine the sequence type

Because the *bona fide* cDNA fragments in the raw sequences with RECA cases are difficult to determine unambiguously, these ESTs should be filtered out and subjected to further scrutiny before their deposition into the public databases like GenBank. Unfortunately, most of them have been submitted to GenBank by conventional EST processing pipelines that do not examine cDNA termini and their variations (Additional file 1: Figure S2 A–J, M–O).

### Double-Termini Adapter (DBT)

**Figure 4:**
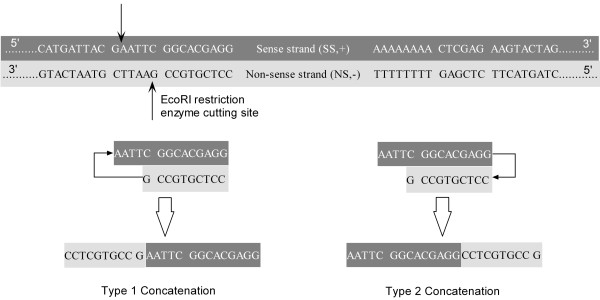
Schematic view of double-termini adapters showing two types of concatenation.

Previously, we reported abnormal ESTs with double-termini adapters (DBT) – a palindrome linker, made from two mutually exclusive terminus components (*e.g.*, the adapter 5′ -CCTCGTGCC- 3′ from 3TNS, the *EcoR*I site 5′ -GAATTC- 3′ from either 3TNS or 5TSS, and the adapter 5′ -GGCACGAGG- 3′ from 5TSS, where 5TSS and 3TNS should be, in theory, mutually exclusive as per Figure 1), that could bring separate 3′ and 5′ directional sequence fragments together into a single, chimeric EST sequence [10,11]. Detailed pattern analysis of these DBT ESTs reveals two distinct sub-categories, which we call Type 1 Concatenation and Type 2 Concatenation, and note that Type2 Concatenation is a novel finding in this study. As shown in Figure 4, Type 1 Concatenation possesses a sequence pattern of CCTCGTGCC + G + AATTC + GGCACGAGG, whereas Type 2 Concatenation has AATTC + GGCACGAGG + CCTCGTGCC + G. Type 2 concatenation indicates that *Adapter1* in the sense strand can be connected directly to *Adapter1* in the non-sense strand. Among 309,976 raw *Pinus taeda* ESTs, we found that 3.5% of the UGALAB ESTs (6,045 out of 172,229) and 0.9% of the NCSUFBG ESTs (681out of 75,001) have Type 1 Concatenation, while 2,515 from UGALAB and 741 from NCSUFBG possess Type 2 Concatenation. It is interesting to note that many ESTs have continuous, repetitive and mixed Type 1 and Type 2 Concatenations, as shown in Additional file 1: Figure S3 A and B. How many repetitions of such combined concatenations exist in a sequence? This might be a random effect involving complex biochemical reactions, because we have uncovered single-, double- and triple-DBT repetitions. Because of such complex concatenations, we might not be able to conclude that all DBTs will bring 3′ and 5′ directional sequences to form a single chimeric EST. For example, as shown in Additional file 1: Figure S3 B, *FLD1_32_F06.b1_A029* has a complex concatenated adapter immediately before the **3TNS** terminus: CCTCGTGCC (*Adapter1* in non-sense direction) is connected to GAATTC (*EcoR*I site); GAATTC is concatenated to GGCACGAGG (*Adapter1* in sense direction); GGCACGAGG is concatenated with CCTCGTCC again, which is part of the normal **3TNS** terminus. In this case, all of the sequences should belong to 3′-directional sequences, not chimeric sequences at all. In fact, about 0.24% of 3′-end sequences of UGALAB have the pattern *5TNS + N + DBT + 3TNS* with this feature. When processing this kind of sequence, it is appropriate to take only the region between 5TNS and 3TNS, excluding DBT, as the cDNA inserts. However, current processing pipelines have overlooked this abnormal terminus and submitted sequences including DBT to GenBank (Additional file 2: Table S1).

### Our software tool - AFST

Current EST processing pipelines that do not examine cDNA terminal structures apparently do not have the ability to detect and filter aforementioned abnormal sequences properly before the GenBank dbEST submission. Using pattern analysis, we have created a unique EST processing protocol to determine the *bona fide* cDNA inserts within raw EST sequence reads generated by Sanger sequencing. Based on this protocol, we developed a software tool called AFST (*A*bnormality *F*iltering and *S*equence *T*rimming for raw ESTs) that can identify cDNA terminal structures, visualize sequence abnormalities, and trim ESTs properly. As an open-source tool, the executable and source codes of AFST are available online (http://code.google.com/p/afst).

**Figure 5:**
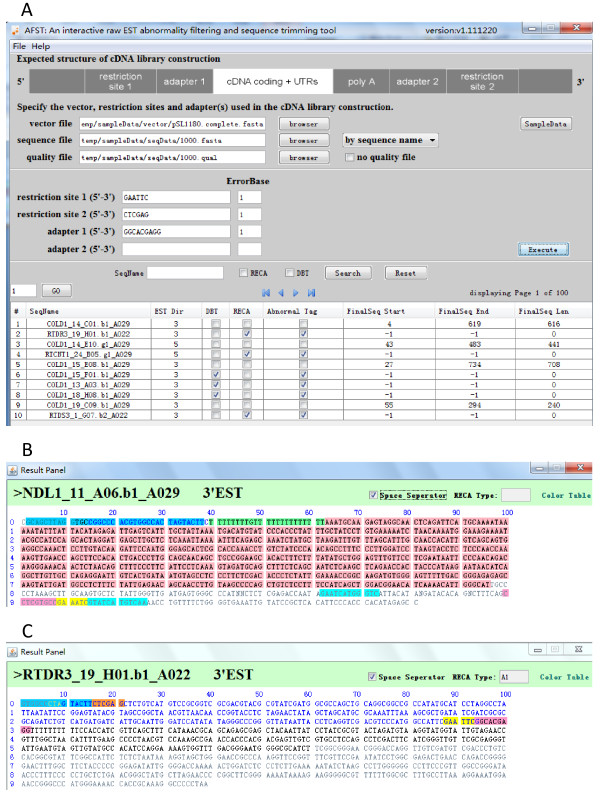
**Snapshots of AFST user interfaces. a**: The main interface allows users to upload their sequences, specify relevant information about vector and adapter/linker sequences, initiate data processing, and obtain tabular results showing abnormality. **b**: Details of a normal sequence. The high-quality region between 5TNS-4 (from 2 to 62, marked with blue and green) and 3TNS (from 900 to 926, marked with pink, yellow and blue) is the final clean sequence (*i.e.*, the region with a light red background). The color legends and their meanings can be found by clicking ‘color table’. **c**: Details of an abnormal sequence. This sequence has RECA abnormality (RECA-Type A1), where the double-stranded cDNA insert is inverted in its orientation and inserted into the double-strand plasmid vector after enzyme digestion. The vector sequence region between 5TNS-2 (highlighted with blue and brown) and 5TSS-1 (highlighted with yellow and pink) is the part that should have been cut off theoretically after enzyme digestion.

Implemented in Java with a MySQL or SQLite backend database, AFST allows users to load their raw ESTs in FASTA format, with or without a relevant quality file, and specify the vector sequence, adapter sequence(s), and the restriction enzyme recognition sites adopted in their cDNA library construction protocols. After execution, as shown in Figure 5A, AFST is able to provide a tabular result showing final clean, trimmed sequences and information about sequence abnormality such as DBT and RECA. Moreover, AFST can provide more detailed information about the cDNA termini (Figure 5B) and abnormalities detected (Figure 5C) for each individual sequence, and allow users to export results selectively for further data analysis. Besides the GUI version, we also create a command line version of AFST that can be easily integrated into existing bioinformatics pipelines for automatic data processing.

In order to compare AFST with other EST cleanup tools or pipelines, we compared Genbank ESTs for two species with our processing results because these Genbank ESTs have been trimmed by other tools or pipelines. Of the 309,976 raw *Pinus taeda* ESTs, we carefully examined 230,783 GenBank ESTs that had been submitted by at least three different EST processing pipelines [1-3,10]. We found that among them 5.2% (*i.e.*, 11,986 ESTs) are “unclean” and 2.2% (*i.e.*, 5,078 ESTs, including 3,180 that have DBTs) are abnormal, both of which could be cleaned or filtered by AFST. Moreover, two other popular tools for raw Sanger EST cleanup and trimming – Lucy [13,14] and SeqClean [15] were adopted to process the 309,976 raw ESTs. Interestingly, among the final clean ESTs trimmed by Lucy, 55,993 (18.1% of 309,976) are still unclean, 3,789 (1.22% of 309,976) have DBTs, and of all the 1,087 RECA sequences, 771 ESTs (70.9% of 1,087) are unidentified. Meanwhile, among the final clean ESTs trimmed by SeqClean, 33,494 (10.8% of 309,976) are still unclean, 6,416 (2.1% of 309,976) have DBTs, and of all the 1,087 RECA sequences, 934 ESTs (85.9% of 1,087) are undetected. In order to demonstrate that our protocol performs well for cDNA libraries other than those in *Pinus taeda*, we reprocessed 38,709 peanut (*Arachis hypogaea*) ESTs from GenBank dbEST that utilizes pBluescript II SK as the vector and *EcoR*I and *Xho*I as the two restriction sites. Consequently, we found 25.3% (*i.e.*, 9,785 ESTs) are “unclean” and 3.9% (*i.e.*, 1,510 ESTs, including 259 that have DBTs) are abnormal using AFST.

## Conclusions

The ever-growing collection of EST sequences in GenBank is an important bioinformatics resource, providing crucial data for downstream applications related to gene identification, functional annotation, SNP and other polymorphism identification, and so on. Providing clean data is crucial if these applications are to be used to correct analysis. Although there have been significant efforts to filter error-prone ESTs, many GenBank ESTs are still problematic. As demonstrated in both this and previous studies [10,11], current bioinformatics protocols and approaches do not explore the essence of potential EST data abnormalities from the perspective of cDNA library construction. Consequently, they have deposited a significant amount of unclean and abnormal ESTs into the public repositories and created potential problems for data-dependent downstream applications. Without inspecting cDNA terminal structures, existing EST processing programs fail to achieve sufficient data quality control and are unlikely to identify and remove common error-prone ESTs before GenBank deposition. In this study, we have adopted a novel pattern analysis approach that proves to be effective in identifying and distinguishing EST sequence abnormalities based on cDNA termini structures. This approach improves identification accuracy of the *bona fide* start and stop position of a cDNA insert within a raw EST sequence, thus significantly improving EST data quality. It also helps illustrate wet-lab abnormalities that can reveal potential error sources, such as a failure of one or both of the restriction enzymes to cut the plasmid vector, a failure of the restriction enzymes to cut the vector at the correct positions, the insertion of two cDNA inserts into a single vector, the insertion of multiple and/or concatenated adapter links, the presence of 3′-end terminal structure in designated 5′-end sequences and vice versa, and so on. In particular, the double-termini adapter (DBT) reported previously as one EST abnormality [10,11] proves to have a novel variation (*i.e.*, the Type 2 Connection) identified by the pattern analysis approach adopted in this study. Clearly, our pattern analysis approach and the relevant software tool AFST will help biologists diagnose the potential problems in wet-lab procedures and facilitate creation of more accurate data.

## Methods

We downloaded all 309,976 raw Sanger ESTs for *Pinus taeda* from NCBI Trace Archive (http://trace.ncbi.nlm.nih.gov/Traces/trace.cgi), which were generated by three different sequencing centers or labs including previously well-known TIGR institute. For these raw ESTs, we were able to collect the complete information about cDNA library construction protocol (*i.e.*, plasmid vector, adapter or linker sequences, restriction enzyme sites, sequence name convention and associated sequencing directions), which is required by AFST to conduct accurate pattern analysis. In particular, 230,783 out of 309,976 ESTs were submitted by each center or lab into GenBank dbEST as final clean ESTs after raw EST cleanup and trimming. Therefore, this dataset represents a valuable benchmark for us to evaluate AFST performance. Also due to the availability of the complete cDNA library construction information, we were able to use two other popular Sanger EST cleanup tools – Lucy [13,14] and SeqClean [15] to process 309,976 raw ESTs for performance comparison with AFST. In order to demonstrate that our protocol performs well for cDNA libraries other than those in *Pinus taeda*, we downloaded all *Arachis hypogaea* (peanut) ESTs (86,939) from dbEST, which were deposited by many labs and investigators. Among them, the biggest data set was 38,709, whose complete cDNA library construction protocol information (*i.e.*, pBluescript II SK as the plasmid vector and *EcoR*I and *Xho*I as the restriction enzyme sites, etc.) was available by extracting and parsing dbEST records. Because cDNA library construction information is not mandatory for dbEST submission, it is often difficult to get complete cDNA library constructional information among dbEST records. Therefore, we used AFST to process 38,709 peanut ESTs and detect cDNA terminus patterns.

In our pattern analysis protocol, there are three important concepts worthy of further explanation: *Pattern*, *Confidence score* and *Reasonable pair*. They are also implemented in our software tool AFST to identify abnormal sequences such as those with RECA and DBT and determine final clean ESTs.

### Pattern

“Pattern” refers to a cDNA terminus structure detected in a raw EST sequence. It is determined by the type, number, order and context of all cDNA termini in terms of the specification (expectation) given by a specific cDNA library construction protocol. To identify the pattern, we first find all putative cDNA termini existing in the sequence, then consider good/low quality regions and vector fragment positions, and finally determine the pattern with respect to the following aspects:

(1) Type of cDNA termini. There are four major types of termini [**5TSS**], [**3TSS**], [**5TNS**] and [**3TNS**] as shown in Figure 1. Each of these termini can be further categorized into some sub-categories. For example, [**3TSS**] includes **3TSS**, **3TSS-1**, **3TSS-2**, **3TSS-3**, **3TSS-4** and **3TSS-5** (also see Figure 1).

(2) Number of cDNA termini. For instance, a sequence with just one [**5TSS**] and another sequence with two [**5TSS**] have different patterns.

(3) Sequential order of cDNA termini. For example, a sequence with first a [**5TSS**] and then a [**3TSS**] has a different pattern from another sequence that has first a [**3TSS**] and then a [**5TSS**].

(4) Context of cDNA termini. Flanking region context refers to the sequence region immediately before/after a terminus. There are mainly two cases: vector fragment (represented by *V*) and non-vector fragment (*N*). Furthermore, vector or non-vector fragments can be further classified into vector fragment in high quality region (*HV*), vector fragment in low quality region (*LV*), non-vector fragment in high quality region (*HN*) and non-vector fragment in low quality region (*LN*). Context is one of the basis on which terminus’ confidence is estimated by computing a confidence score (see below).

### Confidence score

Because of sequencing errors [16-18], some *in-silico* identified cDNA termini might be false positives. When a terminus defined in Figure 1 is detected, we will quantify our confidence in its detection with a confidence score, which is calculated by considering the extent of the completeness of all required sequence elements, adjacent sequence contents (contexts) and the percentage of bases that match the whole terminus.

(1) Determine the completeness score for a given terminus (***A*** score). The completeness score is directly reflective of the number of sequence elements in the terminus. For example, the completeness score of **5TNS**, which has three sequence elements (*i.e.*, a poly(T) tail, *Xho*I site, and *VF2*), is higher than **5TNS-1** and **5TNS-2**, each of which have only two sequence elements. Comparing with **5TNS**, **5TNS-1** and **5TNS-2**, the completeness score of **5TNS-3** is the lowest one because it only has one sequence element. The terminus with the higher completeness score is more likely to be authentic, instead of being an artifact of a sequencing error.

(2) Score a terminus according to its flanking region context (***B*** score). Sequence contents that match the expected structures in terms of a cDNA library construction protocol deserve a higher score. For example, we expect to detect a vector fragment sequence immediately upstream of **5TSS**. Correspondingly, the vector fragment, the low-quality non-vector fragment, and the high-quality non-vector fragment detected immediately upstream of 5TSS will result in the highest (100), intermediate (50) and lowest ***B*** score (0) respectively, which will be assigned to the identified **5TSS**.

(3) Score the percentage of matched bases (***C*** score). The percentage is calculated in terms of the detected bases that are the same as the expected bases divided by length of the terminus. For example, the 3' EST *NDL1_11_A06.b1_A029* in Additional file 1: Figure S3 C has the cDNA terminal pattern of *5TNS-4 + N + 3TNS-5 + N + 3TNS + V*. Obviously the cDNA terminus detected in the front is **5TNS-4**, whereas the terminus detected at the end can be either a **3TNS** or **3TNS-5**. Because the percentage of matched bases is much lower for **3TNS-5** than for **3TNS**, as well as due to the adjacent sequence contents (contexts), **3TNS** is assigned with a higher ***C*** score than **3TNS-5**. Therefore, we identified **3TNS** as the real terminus at the end of this EST while **3TNS-5** was a false one. The formula that determines the confidence score for a given terminus is:

(1) Confidence Score=weight A*A score+weight B*B score+weight C*C Score.

### Reasonable pair of detected termini

Based on the expected cDNA terminus structure shown in Figure 1, if the cDNA insert is short enough, we should be able to detect both the 5′ end terminus and the 3′ end terminus in an EST sequence. This also means that we are able to detect a reasonable pair of cDNA termini for some ESTs, using the following definitions: (1) for a 5′-end EST, [**5TSS**] and [**3TSS**] is the reasonable termini pair where [**5TSS**] should be upstream of [**3TSS**]; (2) for a 3′-end EST, [**5TNS**] and [**3TNS**] is the reasonable termini pair where [**5TNS**] should be upstream of [**3TNS**]; (3) the distance between the two paired termini shouldn’t be too short to contain a cDNA insert (i.e. > = 200 bases).

In our software tool AFST, essentially, we first determine all cDNA terminal patterns according to the type, number, order and context of all expected termini, and identify and filter out RECA and DBT abnormalities. We then search for *reasonable terminus pairs*, calculate *confidence scores* for all detected termini, and select the reasonable terminus pair that yields the highest cumulative confidence scores to delineate the start and end positions of the *bono fide* cDNA insert. Finally, the final clean sequence is obtained by trimming off both low-quality regions and vector fragments from the sequence fragment between the two termini of the best reasonable pair.

## Abbreviations

5TSS, 5′ terminus of the cDNA in the sense strand; 3TSS, 3′ terminus of the cDNA in the sense strand; 5TNS, 5′ terminus of the cDNA in the non-sense strand; 3TNS, 3′ terminus of the cDNA in the non-sense strand; V, Vector sequence, either HV or LV; N, Non-vector sequence, either LN or HN; HV, Vector fragment in high quality region; LV, Vector fragment in low quality region; HN, Non-vector fragment in high quality region; LN, Non-vector fragment in low quality region.

## Competing interests

The authors declare that they have no competing interests.

## Authors’ contributions

GJ and CL managed and coordinated the project. SZ carried out main implementation and data analysis. XL, PL and JM contributed to some implementation. JK contributed critically to manuscript editing. All authors participated in manuscript writing and editing. All authors read and approved the final manuscript.

## Supplementary Material

Additional file 1 **Figure S1.** Sequences with abnormal cDNA terminus structures. All the sequences mentioned in **Results and discussion** section (Part 1) are listed. **Figure S2.** All types of Restriction Enzyme Cutting Abnormity (RECA). All sequences that possess RECA and are described in **Results and discussion** Section (Part 2) are listed. **Figure S3.** Other examples of abnormal EST sequences. All other abnormal sequences discussed in this paper are listed. Click here for file

Additional file 2 **Table S1.** Some raw EST sequences with the “5TNS + N + DBT + 3TNS” pattern had been submitted to GenBank. Most of them have their DBTs untrimmed in Genbank. Click here for file
