# Supplementary material for: Pattern analysis approach reveals restriction enzyme cutting abnormalities and other cDNA library construction artifacts using raw EST data
Source: BMC Biotechnol. 2012 May 3;12:16. doi: 10.1186/1472-6750-12-16 (PMC3424822; doi:10.1186/1472-6750-12-16)
Supplement: Additional file 1 — Figure S1. Sequences with abnormal cDNA terminus structures. All the sequences mentioned in Results and discussion section (Part 1) are listed. Figure S2. All types of Restriction Enzyme Cutting Abnormity (RECA). All sequences that possess RECA and are described in Results and discussion Section (Part 2) are listed. Figure S3. Other examples of abnormal EST sequences. All other abnormal sequences discussed in this paper are listed. [file 1472-6750-12-16-S1.pdf]

- **Supplemental Figure 1 (Figure S1)**

  -- Sequences with abnormal cDNA terminus structures
- **Supplemental Figure 2 (Figure S2)**

  -- All types of Restriction Enzyme Cutting Abnormity (RECA)
- **Supplemental Figure 3 (Figure S3)**

  -- Other examples of abnormal EST sequences

All three supplemental figures share the same color legend: (1) font color: dark black for high-quality sequence region, gray for low-quality sequence region, blue for high-quality vector region, and gray-green for low-quality vector region; (2) background color: blue stands for vector, yellow for *EcoR*I, brown for *Xho*I, pink for adapter1, green for poly(A)/(T), and light red for our final clean sequence.

Supplemental Figure1 (S1)

>gi|57885292|COLD1_45_G11.g1_A029 Case No. 1; EST Dir: 5’; Pattern: N,3TSS-3,N,…3TSS-3,N **A**

```
           10         20         30         40         50         60         70         80         90        100
 0 ACTATGGCAA GTGGGATGAT CATATGCAGC AAATGGCTAG CTTTTGGAGA TGAAGAGCAA AAATGACCAC GGCGTCGACT TCTGGAACGC CCCGGAGCGT
 1 TCAGGATGGT TGATGAAGCA GGGCGAGTAC ATCAAAACAT GGAGGCGCAG ATGGTTTGTT CTAAAGCAGG GAAAGCTCTT CTGGTTCAAG GAAAATTACA
 2 TCACAAGGGA TTCTAATCCC CGTGGTGTTG TTCCGGTGAG CACCTGCTTG ACTGTCAAGG GAGCCGAAGA CGTCCTCAAC AAGCCATTCG CCTTCGAGCT
 3 CTCGACGAGC AGAGAGACCA TGTACTTCAT CGCAGACAGC GATAAGGAGA AGGAGGAGTG GATCAATTCC ATCGGCCGCT CCATCGTACA GCATTCCAGG
 4 TCAGTTACAG ACAAGGAGAT CGTTGATTAT GATAGCCAGC GTGCCGATAA ATGAATACCC AATTCGAATC GGATGGATTC GCTGTAAATT GGTTGCAATT
 5 AGGGTTTCTA GGGTTTTCTT TTGGGTTTTG TGATGGAACG CCTTAAATCG GTTGTCCATT GCATTTCTAG GATGAATTTT AATAAATTTG TATGAGATCT
 6 CTAGCTGGGC TTTTATGGAT TCTAGCAGCA GAGGCTTTTA GATGTACAAA GCCTTTGACC TGTATAGTAA ATAACAGCAA GAGTGGTTTA AACTTTGTAA
 7 TTTCAATTTG GTGATGGAGG TTGAAAATGT TGGATATATA TCANANAAAA AAAAAAAAAA AAAAAAANAN AANAAAAAAA AAAAAAAAAA AAAAAAAAAA
 8 AAAAANNAAA AANAANANNN AAANNNNNNN NAAAAATTTT ATTGGCCCCT GGGGGCCGGG CCCATAATTT GGGGGGGGGG CCGTTTTTTT AAACCTTTGG
 9 AGCGGGGAAA ACCCGGGTTT TCCCAATTAA ATCCCCTTTT GAAGAAAACC CCCTTTTTCC ACCGGGGGGA AAAATAAAAA AAGGGCCCCC CCAATTTCCT
10 CTCCCAAAAT TTTTCCCCCC AAAAAAGAAA AAGGGCCCCC AAAGGCGATT TTTTTCTACT CCCCTTGTGG GGGGATTTTC CCCGCCAAAC TCTAAAAACA
11 CATTTATTCC CCCCTGATAC GGCCAAATAA ACGGGGGGGT GGTGGGTTTT AACCAAAGTG TACCCAAAAT TTTCCACCCC CAAGCCCCCC CTTTCTTTTT
12 TTTTTCTTTT TTATTTCCCC AGATTGGGGT GTTTCCTCCA AAATAAATAG AGGGGGCCTC CCTTGGGGGC AAAAATATGT TCTCCGGCCC CCACCACAAA
13 TATATATTGT GTGAGTGTCA CTTGGGGCCC ACCCCCTAAT AAGTGTTTCC TTTTTA
```

The Genbank submission for this sequence is from 32 to 766 (see http://www.ncbi.nlm.nih.gov/nucest/57885292), with polyA not trimmed off whereas our final clean sequence is from 32 to 741.

>gi| 57881876|COLD1_13_D11.b1_A029 Case No. 2; EST Dir: 3’; Pattern: (N,)5TNS-4,N **B**

```
           10         20         30         40         50         60         70         80         90        100
 0 AGCTTCGGCC AGTGGCCCTA GTCTTCTTTT TTTTTTTTTT TTTATGGCAA AATACTCATA ATTGGCATTC AAAATAATCG TTATTAAAAT GACAACCAAC
 1 TTCTGATGAC TTTTGAAAGC ATCAAATAAA ATCTCAAGTT CTACTGTTGA CTACCAACAT GGCAACCTCA AATTGAAATT TCCATATAAT AACATGAAAC
 2 CACACTTATA CAGTCAGAAA ACTTGTACTT CTGAATTTCT CTAAGAAGAA ACTCTAACTT GATCTTCTGC ATTCAATAAC CTTGCACCTT AGTAAAACTT
 3 CACAATGTTA TGCCTCACCA GCAAACAGGA GAATCTTGCA TAAATACCAT GGGATGTTTT CCCAGCACAC TTAACTTTGG ATAACCAAGT GCATCGATTT
 4 CTTGATCCTC AAAATCCAGC CCATCCTGCA GGTTGAGATT GAGTTATAGA TCGTGAACTT CGGCCATCAG ATTTTGAACT GTATGAATCA TAAACTGCTG
 5 GATTACCCTT GCTTTCACTA CCAGAAGACT TAGATGCCTG TACATTATTA CGAACTGGAG TGAAACTATC CTGCACAAAA CCCCAAGTGT CACTGGAACC
 6 AACACTTGAA CCACTTGCAT GCTGCTGAGG GCGGTAGCCA TTTGAGGCTC CAGACACATT GGATGGGGGA GAATTTGACA ACTGTGTGGT CAGTGATTCA
 7 CCAAATGGTC GCCATTCCTT CGCATCAGTG GCCTTGAAGG GCACGCCATG GGAGGACATA GTAGACATAC CTTCTTGCAG TTCCCAAATA GATCCCTCTA
 8 TCTTGTCTTC TTGCTTGTTG CCTGAGTGCC AGCTTCCAGG GAACCACCGC TGTTTTTGCT GTTGTTTCGG CTGTTGCACT TGTGCCTGTT GGGGTACAAC
 9 TGGCTTTGTG CTACCTTCTT TACCGAATGG GGTGAAGTTC GAACAATTGC TGTAAACAGG TCAGTTTAAC TCCTGAATCT CTTGCTTTGA GCACGGCAAA
10 AAGCTGCAAA TTTTCAACTT TGAGGTAATT CGGCCCTTTT TAACGCACTG CTCGACTCTT CTTCAATAAT CCCATTCTAC TGGAAACTTT TTTGCCCAAC
11 CACTATTCAA TGGGGGGTTA TGGTTAAAAT TCGCAAAAAG TCTTTAAAAA ATCTCTAAAA GGCCCCAATT TTTAAACTAA ACTTCCGGGA ATAACCAAAC
12 CATTGGTTTC CCCCCGGGCA TTATCCAAAC AAACCCTATG GTTTATAAAA TAATTTACCA ACCGGCAGAT ATAGGTGGGG AGACACCTTT AAAAATTGAA
13 ACCCTTTAAA AGCTCCCCGA GAATTTTTGG AAAAAAACAA TTATAAATTA GGGTTTACAC TGTAA
```

The Genbank submission for this sequence is from 46 to 821 (see http://www.ncbi.nlm.nih.gov/nucest/57881876, a reverse complimentary sequence had been submitted).

>gi|57884024|COLD1_33_C08.g1_A029 Case No. 8; EST Dir: 5’; Pattern: N,3TSS-4,V **C**

```
           10         20         30         40         50         60         70         80         90        100
 0 NNNNCCATGT CAAGAAGATG GCTTGGAGGG AGAGTGGCCA TTGTAACGGT GCCTCCAGGG GGATTGGTAG GGAAATTGCC TTCCATTTGG CGGAAAAGGG
 1 AGCCAAAGTG GTCGTCCATT ACTCCTCCAA TCAAGCCGCG GCGGAAGAGG TGGCCTCCAA AATCAATAAT TCGGCTTCTT CCGGTGACGG TGTTCGGGCG
 2 ATTGTGTGCA AAGCCGATGT GGCAGAGCCC TCCCAGGTCG CCCACCTTTT CGACACGGCT GAGCAATCGT TTGGGACCCT CCACATCGTG GTAAACAATG
 3 CAGGAGTGGC GGACTCAAAC TATTCCTCCC TCGCACAGAC CTCCGATGAG GACTGGGACA GGATTTTCCG AGTGAATTGC AAAGGGGCTT TTCTTTGTAG
 4 CAGAGAAGCT GCTAACAGGG TTGTCCGCGG TGGCGGTGGC CGCATCATCA ACCTAAGCAG CTCCCTGGTG GGGTTACCGA CACCGGGGTT CGGCGCCTAC
 5 ACGGCCAGCA AGGCGGCGGT GGAGATGATG ACGAGAATCC TGGCACAGGA GTTGAGGGGC ACTCACATTA CTGCAAATTG CGTGGCGCCA GGCCCTGTTG
 6 CCACTGATAT GTTCTTTACC GGGAGGAGCG CGGCGGCGGT GGAAGCGGCG GCCAAATCCA GCCCGTTTGA TCGGTTGGGG AAGGTGGAAG ACGTGGCTCC
 7 GGTGGTTGCG TTCTTGGCGA GCGATGAAGG AGAATGGGTT AATGCTCAGG TCGTCCGGGT CAATGGAGGC CGGGTTTAAT TCATAAACTG GATGGTTTCT
 8 CTCCACATTT TATTTTTTCT GGATTATTAT ACTGTGCTTT CAGGCTTCAC TTGGGGTTTT AATGGAATGT GTTCACTCTT GTCTTTCAAT TAAGATAAAG
 9 TAAATATTTA GCATATATTT AAATTTCTGC TATGGCTAAA AAAAAAGAAG TTCTAGGTGG CCACGTGGGC CGTGCCCCTT AAACTTGGCA CTGGGCCGTC
10 GTTTTACAAC TTCTGGACGG GGAAAACCCT GGCGTTACCA ACTTAATCGC CTTTCGGCCA TCCCCCTTTC CCCGCTGGGG ATATACCAAG AGGGCCCCCC
11 GATTCCCCTT TCCACAAGTT TCCCACCCTG AAGGGAAAGG GCCCCGAATG GGTTTTTCTC CCTCACCTCT GTGGGGTTTT CCCCCCGCAA CGTAAAACAA
12 CCTATTATCC CCCTCTTGGG CCACTAAGAC GGGGGGGGGT GGGTTTTCCA CAGGTACATT AATTTTCAGC CCTTAGCCCG CTCCTTTTTT TTTTTTCTTT
13 ATTCCCATTC CGGGTTATCC CAAAAATAAG GGGGGCCCTT TGTGGCAATA TATGCTTCGC CCCCCCACAA ATTTATTGGG GGTGTCTGTG GGCGCCCCCA
14 AGATTTTCTC TTCTGTGAGC GTCTATATTT GTCGACCATA CAACACACTG CGTTGTTGTA TGTGTGTCT
```

The Genbank submission for this sequence is from 36 to 850 (see http://www.ncbi.nlm.nih.gov/nucest/57884024).

D

>gi|57881078|COLD1_8_E12.b1_A029 Case No. 3; EST Dir: 3’; Pattern: (N,)5TNS-5,N

```
             10         20         30         40         50         60         70         80         90        100
0  AGCAGCTTAG GTGCACGGCC CACGTGGCCA CTAGTACTTC TTTTAGTTGA AGATATTTCA TATCACGAGT AGGTTAAAAG TGGTGAACTC CACTGTTTAG
1  CAAACAATTA CATTCTACTC TTTGGTACGG CGCCCAACTC CTTGGCCCTC GACCTTAGAA TATGCTTTTG GATTTTGCCC GTGGCAGTTT TGGGCAAAGG
2  ACCGAACACC ACCGACTTGG GTCCCATATA ATGGGGGAGA GCAGACCGAC AATAATTCAT TATATCTTGC CCCACACGAC TTTCATCCCC ATTGCGATCA
3  GCAGACCCCT TCAGGGTTAT GAAGGCACAC GGAGACTCAC CCCAACGTTC GTCGGGCCTT GCGACAACTG AGGCTTCCAG GACCATAGGA TGAGTATACA
4  GCACGGTCTC CACTTCGAGG CTGCTGATAT TTTCCCCGCC GGAAATAATA ATGTCCTTTG ACCGGTCCTT GATTTCGATA TAACCGTCTG GGTGCTTGAC
5  CGCCAGGTCG CCGGAGTGAA ACCACCCGCC GCGAAAGCTC TCCCGGTTGG CCTCTGGGTT CCTGAGGTAG CCCTTCATCA TCATGTTGCC ACGCATCACA
6  ATCTCCCCGA GAGTGCTCCC GTCCGCCGGG ACCGGTACCA TGCTCTCCGG ATTCACCACA TCCAGCCCTT CCAGGGAGAC GAAACGCACG CCCTGCCGGG
7  CCTTTAGCCT CGCTCGATCC GGCGGCGGGA GCGAGTCCCA CTCCTCCTTC CACGCGCAAA TGACTGACGG CCCGTTGGAC TCGCTGAAGC CGTATGTGTG
8  CGTCACGTGG AAGCCCTGCT CTTTCATCTT TGCCAGCACA GAAGCCGGAA GCGCCGAAAC GGCNGTCATC GCGTTTACGC CGCGCGGGAT GGAGACTCGA
9  TCCTTGGGTC TCGAGTTCAC TATTGCGTTC AA
```

The Genbank submission for this sequence is from 42 to 781 (see http://www.ncbi.nlm.nih.gov/nucest/57881078, a reverse complimentary sequence had been submitted).

E

>gi|57882944|COLD1_26_D04.g1_A029 Case No. 4; EST Dir: 5’; Pattern: N,3TSS-5,V

```
             10         20         30         40         50         60         70         80         90        100
0  NNNNCATATC TAAGCCNTGC TATTCGGACA CGATCAATGC CTATAGGCCA GCCCTGATTG TGGATTCTGT GCATCTGGAG GTAACCAGTT GTTGCCAGGA
1  GCCTGTTTAC TGTCAAATTC AACAGTGAAG CATGTTTGTG AAGGTGATTC TCGACCATGG TTTACCCGAG GATGCCCAAG CCAATATGGT TGGCTGGCTG
2  TTCTTGGACT GGCACTGTAC ATTATTTTCT TTGCTCCTGG AATGGGAACA CTTCCTTGGG TCATCAACTC TGAGATTTAT CCTTTGAGGT ACCGAGGTAT
3  CTGTGGAGGC CTGGCTGCAA CAGCAAATTG GGTTTCTAAT TTGATAGTGG CGCACACATT TCTCACTATG ACCGTAACCA TTGGCACTTC CATGACATTC
4  TTAGTTTTTG GGGTAATATC AGTGATTGCA CTTTTCTTTG TACTCATTAT TATGCCTGAA ACAAAGGGCC TTTCACTTGA GCAGATTGAA GGGACGTTGC
5  AACAGATAGT AGAGAATTTC AGCTTAAAGT TTTGGAAGAC AAATAACCCC ACAGTTTCTT CTAATAAAGA AAACCTCATA AACAATGATC TTGCAAGCTA
6  GCTATTGTAC GTCTCTACCT GTGAAATAAC TGGATGCATA GGGATATAGT ATCCTAAGAT CCTGTTGGAG AAATTCCAGT GGGTGTATAT TATGCCAAAG
7  GTATTGAATC CGACAAGAAA TGCTGTTGTA GATTTCTCCA CACAAGACAT TTGAGCTCTC GACATGTATA GATGAACAGT ATGGTCTCGG AGTGGACAGT
8  TTAACTCTCT ACATGTGTCA AAAATAAGTA TCCCTATGGT TCCAGATTAT GAAAAAAGAA GTACTAGTGG CCACGTGTGC CGTGCACCTT AAGCCTGGCA
9  CTGGGCGTCG TTTTACAACG TCCTGACTGG AAAACCCTGG CGTTACCCAA CTTATCGCCT TGCATCAAAT CCCCCTTTCG CCGCTGGGTT AATACTAAAA
10 GGCCGCACCG ATTGCCTTCC AAAGAGTGCC AACCTGAATG GCAAGGGCCC CGGTGCGGAA TTTCCCCTAC GCTTCTGTAG GATTTCCACC GCGCCTTTAA
11 GCCACCATAA TCTGCCCGGT AGGGGCAATA AACCGCGGGG GTGGGGGTTA TCCCGCGTAA CCCCAACTTT GTCGGGCCTA AGCCCCTCCT TTCGTTTTTC
12 CCTTTTTTCC CCACTTAACC GGGTTCCCGT AAGTTAAAAG GGGTCCCTCT GGGTCCCATT AGGTTTGGCC CCTCCCCAAA ATGTTTGGGA GAGTTCCATG
13 GGCACCCCGA AAAGATTTTC CTGTAGGAAC ACCTTATAAG AGACTTTCAG AGAACCAACA CGGGTTTTTT ATTAGTTTAC CCCCTGTTAA AAGTATAAAA
14 ATCTTAATAT AATTTGGGTG ACATTATACA ATCCTCACCC CACAAAGGTT TTTCCAATTT GCATATACTC TTACTG
```

The Genbank submission for this sequence is from 55 to 762 (see http://www.ncbi.nlm.nih.gov/nucest/57882944), similar to our clean sequence.

F

>gi|48943026|NDL1_12_C01.b1_A029 Case No. 6; EST Dir: 3’; Pattern: 5TNS-2,N

```
             10         20         30         40         50         60         70         80         90        100
0  CGCAGCTTAG GTGCACGGCC CACGTGGCCA CTAGTACTTC TCGAGACTAG TTCTCTCCCT CGCCCATCCA TGTGGACGCC GCGAGCGGCG GATTTATTGC
1  ACCGTTCCTG TACCCTGAGT TGGAATGGGA TTTCCGGCTT CCGTTGGTGA AGAGCATCAA CGTCAGCGGG CACAAGTATG GTCTCGTCTA TGCCGGAATC
2  GGATGGGTTA TCTGGAGAGC AAAGCAAGAT CTCCCTGAGG AGCTCATCTT TCACATCAAT TATTTGGGCG CCGATCAGCC TACCTTCACC CTCAATTTCT
3  CCAAAGGTGC CAGCCAAATA ATTGCGCAAT ATTACCAGCT CATACGTCTA GGGTTCGAGG GCTACCGGAA TATCATGGGC AACTGCGCGG TTAACGCCAA
4  GGCTCTGTCA GATGGTTTGG TGAGGACCGG GCGGTTCAAC ATTCTATCGA AGGAGATCGG GGTCCCTCTG GTGGCGTTTT CGCTAAAGGA CAGTAGCAGG
5  CACGATGAGT ATGAGATTTC TGACCACCTG AGAAGGTTTG GTTGGATCGT GCCCGCGTAC ACCATGGCTC CCGACGCACA AGAGGTGAGG CTTCTGCGTG
6  TGGTGGTTCG CGAAGATTTC AACCGGAGCC TGGCAGAACG ACTGGTTCAT GACATCGAGA AGGTGTTGCA TGAACTTGAC GCGCTTCCTT CAAAGATAGC
7  GAAGGGAAGT GGTTGCGTCT CTGGTAGATG GCCATCCAGA GCTCAAAGAA GGTCAAGACC TAGGTATAGA TGTCACCCAG TTTAAGTCCT CTGCTGTGTT
8  CAACGAAATC GTCAACTCCC ACAAGGCGGT AAAAGCATGT GAAGAATTTG TTGCGCANAA AGCCAATCGT GTTTGCTGAG GAGCCCCATT CAAATGCTGT
9  AAGTTTGAAG TGTTAAAATT ATTTTTGTTT TGTN
```

The Genbank submission for this sequence is from 46 to 736 (see http://www.ncbi.nlm.nih.gov/nucest/48943026, a reverse complimentary sequence had been submitted).

G

>gi|34490922|RTWW2_3_H01.g1_A021 Case No. 7; EST Dir: 5'; Pattern: N,3TSS-5,N

```
          10         20         30         40         50         60         70         80         90        100
0 ATATCTATTC TCGACTAGGC TTCTAAGCGA GGCGCTTTTA GCTGATCCAG GGCTGAAATA CCTTCTTAAC ATTGGTTCAA AGCATCTGCG GGAGCACTTT
1 CGGTTGCCAG GAGCTTCATA GCAGTTGTGA GAGTGGTGAC TAAAGTCGAT TAACCATGTT TGGGCGTGCG CCAAAAAAGA GTGACAACAC AAAATATTAT
2 GATATTTTGG GTGTGTCAAA AAGTGCCACA CCTGACGAGT TGAAGAAGGC TTATAGAAAA GCTGCCATCA AAAATCACCC TGATAAGGGT GGGGATCCAG
3 AAAAATTCAA AGAATTGGCT CAGGCTTATG AAGTCTTGAG TGATCCAGAA AAAAGGGAGA TATATGATCA GTATGGAGAA GATGCCCTGA AAGAGGGAAT
4 GGGAGGAGGT GGTGCTTCCC ATAATCCCTT TGACATATTT GAGTCTTTCT TTGGAGGTTC TTTTGGAGGA TCTAGTTTTG GAGGCGGCAG CTCAAGGGGC
5 CGAAGGCAAA AGCAAGGTGA AGATGTTGTA CATCCTCTGA AGGTTTCTCT GGAGGACCTG TACAATGGAA CATCCAGAAA GCTATCACTG TCGAGAAATG
6 TTATATGTTC GAAGTGTAAG GGGAAAGGCT CTAAATCTGG TGCTTCTGGT CGATGTATGG GATGCCAAGG TTCTGGTATG AAGGTCTCTA TTANGCAACT
7 GGGTCCTGGC ATGATACAAC AAATGCAACA TGTCTGTCCT GATTGTAGAG GCTCAGGCGA GAACATCAGT GACAAGGATA AGTGTGGTCA GTGTAAAGGG
8 AAACAAAGTG NTCAAGACAA GAAAGTACTA GAGGTACACG TCNAGAAAGG GATGCTGCAT GGGCAGAAGG ATAGTGTTCA GGGTGAAGCT GATGAAACGC
9 CCGACACTGT TACTGGGGAC ATTGNTTTTG TATTGCAATT GAAGATCACT C
```

The Genbank submission for this sequence is from 43 to 744 (see http://www.ncbi.nlm.nih.gov/nucest/34490922), same to our final clean sequence.

H

>gi|66977087|RTCA1_11_E04.g1_A029 Case No. 5; EST Dir: 5'; Pattern: N,5TNS-1,N

```
           10         20         30         40         50         60         70         80         90        100
0  NNNTTAGACG CGANATACTA TTTTATTATT AAAAGACATA AATTCTTGAC ATGATGTCCA GAAATATCCC CGAGCAATAT TTATTTAGTT TGTAAGATGC
1  CTACAGAATG CCGGGATTTA TTTAGTTTGT AAAGTGCATA CGGGATGCTT AGCCCAACAG CTCAATAGGG CATGAAAATG ATATGGTTCA AAGGGAGATC
2  ATACACTATG GAACATGTAG GGCACTACAT CAATGACTCA GCCCAATGTA TTGCAGGCCC GCTGCGTGAA GCCCAAACGA TTCTTTTCTC TGTCATACAA
3  TATATAGAAG TTGTTCTGCT GAATGTTTCC TATGATAACG GCAGGTCCAT GAACAAAAGT GCCACCATTC ATCGCCAAGC AGAACGTATC AGCACTACCA
4  GAAGTTTGAG AAGAGAAACT ATTCTCACTA GGAACTACCA TGTCTGAGCC ACCCTTGAAA TGAAATGCAA ATGTAGGCCA CTGAAAATTC TCTAAACCAG
5  ACACATTGTA ACACAAGTCT TCAACTCTCG AGTTTTTTTT TTTTTTTTTT TTTTGCAGTA TCATTGTATC ATATTAACAT AAAACAGGTA CAATAAGAAG
6  AACAACAATT TGAAACATTG AAGGCTCTAT GCAATGGCCA CCTGACTGGA TATTATACTC AAATGCTTAT AACAACATCT TAAACGTCTA CTGCAACTCT
7  TTCGATAATT GAGAACTAAT TTTTAGTTAA GTTTAAGCTA TGAAGTGAAT TTCCATCAAT GTCATAGAAT ACAGCATTAA CAAACGTTGC TGTAATTTGC
8  ACAAACATGA ATCCTTGACC AGCAAATAGA AATTCAATCC ACCCTGTTTG CATTTGAACT TACTCCCTCC CCAGCTTTTG AACCCCCCCA CTGGTCAGAA
9  AAATGCAGGA ACTTGTCCTG GCTTTTAAGT CCTCCAAACA AGGGGATGCC CCTTTGATTA CAAATCTCCT TTGTTGGCCT CAAAATGGGC CCCACCTGTT
10 GTGAGCCCCC CCCTATCCCC CTGTGTTCCG ATCTTCCGAT GGGGAGAAGA ACCCCATCTA TTTTTCGGGT GGCCCTTCAA CATTTATGGG GTTGTGGAGG
11 CCCCCTAGTT TTTTTTAAGG GAGCGTTAAA AGGGTAAACC CCCCCAACTA AATTTGTTTG GTGGGGCCCA ATATATCCCA AAGGGGGTTG GTCTGGAAAA
12 AAACCCCGAA GAAACTCCTC CTCTTCAGAA AAGGAGGTTT GTGTAAACTT TTTTTTCCCG GGTTTTAATG GATATTGTTG AAGTCTCTCC GCTGGGTTCC
13 AAACAATGCC GGTTTGTGGG CCCCTGGATT GAAAAAATGT CGATTTTTCT TGTTCACATC CCCCATACAT CCTAAGACAA ATTTGCAGGT ATTTG
```

The Genbank submission for this sequence is from 31 to 679 (see http://www.ncbi.nlm.nih.gov/nucest/66977087), not noticing the presence of a 3'-end terminus in this 5'-end sequences whereas we suggest not submitting this sequence as it is abnormal.

Supplemental Figure 2 (S2)

A

>gi| 48933478|FLD1_38_A06.g1_A029 RECA-Type: A1; EST Dir: 5’; Pattern: N,3TNS-1,V,3TSS-2

```
           10         20         30         40         50         60         70         80         90        100
 0 GGGGGTTTCG GCTGATCAAC TACTACAGTA TATAATTATG ACGGCCATTG CTCAAAGAAA GAGCGTGGCA AATCCAGAAA GGATAGCCAT GAAGATGGCT
 1 GCATCTGATG AGACACTGTG AGAAGCACCA GACTTGCTGT TGCTCGCACC TGGGGCGCTT GCTGAACTGT TTGGTGTGGC AGAAGACGTG CTCGATCCGG
 2 GAGCCGGAGA CGAAGCGGAT GTAGCCGGAG AAGACACAGG AGCCGGCGAT GGCGAACTGC CGGGAGCAGC TGCAGTGGAG ACCTTGATGC TCACCTTCTG
 3 TCCGGCGGCA CAGTGGCCGG AAACTGCACA GATGTAGTAA TGGTGGCCGG TGGTGTTGAG AGTGATGGAG GCCGGGCCTG TCTCGTATTT TTCCAGGGGG
 4 CTAGTCGTCA CGCACTTGTC GTAATCCGCC TTGCTCACTC GATAAACATT GTGCACATTT GTAGCGAATT TGAACACGAG GATATCACCG AGCTTAAAGG
 5 TTCTGGCCTT AACCCAGTCG GTGTAGAGCT TGGCATTGCT GGCGGGAATA GTCCATCCTG TGTTTCCGCC CACGGCGTAG GTCGTTGCGG CCACGCTCTG
 6 CAGAACGGCT AGAGCAAGGC ATGCCCCCAA AGCTACAAGA ACCTGTCCTC TTACAGCTGC CATGTCTGTG TCCCCAGGCC GACCCAATAC CCAACCCTAT
 7 CACCTGCGGC AGCGTCCCGT TCTTGACTCG CTGCCTGTGA AACTTTATCG CACAGCAGAG AAGAGCAGCG CAGCGCAGAA CAAAGCAGCA AAGCACCCTC
 8 GTGCCGAATT CGAATGGCCA TGGGACGTCG ACCTGAGGTA ATTATAACCC GGGCCCTATA TATGGATCCA ATTGCAATGA TCATCATGAC AGATCTGCGC
 9 GCGATCGATA TCAGCGCTTT AAATTTGCGC ATGCTAACTA TAGTTCTAGA GGTACCGGTG TTAACGTTAG CCGGCTACGT ATACTCCGGA ATATTATAGG
10 CCTNAGATGC ATATGGCGCC GCCTGCAGCT GGCGCATCGA TACGCGTACG TCGCGACCGC GGACATGTAC AGAGCTCGAG AAGTACTAGT GGCCACCTGG
11 GCCGGTGCAC CTAAACTTTG CCACTGCCCG TCGTTTTACA CGTCCGGACT TGGAAAAACC TGGGGTTACC AAACTTAATC CCCTTGAAAA AATCCCCTTT
12 CTCCCGCTGG GGAATAACCA AGAAGCCCCG ACCCATTGCC CTTCTCAACA TTGCCCACCC CAAAGGGAAA GGGCGCCCAA GGCGATTTTT CTCTTTACCC
13 CATGGGGGG
```

The Genbank submission for this sequence is from 34 to 811, including a adapter/linker fragment (see http://www.ncbi.nlm.nih.gov/nucest/48933478) whereas the cDNA strand in this sequence is not sense but non-sense and we suggest not submitting this sequence as it is abnormal.

B

>gi|34351232|RTDR3_19_H01.b1_A022 RECA-Type: A1; EST Dir: 3’; Pattern: 5TNS-2,V,5TSS-1,N

```
           10         20         30         40         50         60         70         80         90        100
 0 GTGGCCCTAG TACTTCTCGA GCTCTGTCAT GTCCGCGGTC GCGACGTACG CGTATCGATG GCGCCAGCTG CAGGCGGCCG CCATATGCAT CCTAGGCCTA
 1 TTAATATTCC GGAGTATACG TAGCCGGCTA ACGTTAACAA CCGGTACCTC TAGAACTATA GCTAGCATGC GCAAATTTAA AGCGCTGATA TCGATCGCGC
 2 GCAGATCTGT CATGATGATC ATTGCAATTG GATCCATATA TAGGGCCCGG GTTATAATTA CCTCAGGTCG ACGTCCCATG GCCATTCGAA TTCGGCACGA
 3 GGTTTTTTTT TTCCACCATC GTTCAGCTTT CATAAACGCA GCAGAGCGAG CTACAATTAT CCTATCGCGT ACTAGATGTA AGGTATGGTA TTGTAGAACC
 4 GTTTGGCTAA CATTTTGAAG CCCCTAACGT CCAAAGCCGA ACCACCCACG ACGAGTTGTC GTGCCTCCAG CCTCGACTTC ATCGGGTTGT TCGCGAGGGT
 5 ATTGAATGTA GTTGTATGCC ACATCCAGGA AAAGTGGTTT GACGGGAATG GGGCGCATCT TCGGCGGGAA CGGGACCAGG TTGTCGATGT CGACCCTGTC
 6 CACGGCGTAT TCGGCCATTC TCTCTAATAA AGGTAGCTGG GAACCGCCCA AGGTTCCGGT TTCGTTCCGA ATATCCTGGC GAGACTGAAC CAGACCGGGG
 7 GAACTTTGGC TTCTACCCCC GGAGATATTG GGGACCAAAA ACTGGATCTC CCTCTTGAAA AATATCTAAG CCTTGGGGGG CCTTCCCGTT GGCCGGGATA
 8 ACCCTTTCCC CCTGCTCTGA ACGGGCTATG CTTAGAACCC CGGCTTCGGG AAAATAAAAG AAGGGGGCGT TTTTTGGCGC TTTGCCTTAA AGGAAATGGA
 9 AACCGGGCCC ATGGGAAAAC CACCGCAAAG GCCCCCTAA
```

The Genbank submission for this sequence is from 313 to 553 (see http://www.ncbi.nlm.nih.gov/nucest/34351232, a reverse complimentary sequence had been submitted) whereas the cDNA strand in this sequence is not non-sense but sense and we suggest not submitting this sequence as it is abnormal.

C

>gi|34345996|RTDR1_20_F07.g1_A015 RECA-Type: A2; EST Dir: 5’; Pattern: 5TSS-1,N,V

```
           10         20         30         40         50         60         70         80         90        100
 0 TTATCATTCG GACGAGGGCG CCATCTCCAT CTCTTGTTGT ATTATCGTAT TTTCTTGCCT TCTCCTTCAC CTTCACCTAG CTCAGGCGTG AGAATGGCTT
 1 CCGCTGTGCA ATGTGCCGCC GTGAGCAGCC GCCGCGAGCT TGAGGACGTG TTCCGCAAGT TCGACACCAA CGGCGACGGC AAAATATCCA AGTCGGAACT
 2 GAGTGCCCTG ATTTCGGAGG CGGAGATTGA AGGGGTGATG AAGGAGGTGG ACTCCAATAA AGACGGATTC ATCAACTTCG ACGAGTTGGT GGAGGCCAAC
 3 TCCAAGAACC TCAACGCTGC CCGTCTCATC CGAAACTCCG CTTCCGCTGT GCAATGTGCC GCCCTGCCCG GCCGCTTGGA GCTGGAGGAC GTGTTCCGCA
 4 AGTTCGACAC CAACAGCGAC GGCAAAATAT CGAAATCTGA ACTGAGCGCC ATCCTCAAGC GCAGCTGTAG TGAGGAGGAG ATTGATGGTG TGATGAAGGA
 5 CGTGGACTCC AACAAAGACT GCTTCATCAG CTTCGACGAG TTCGTGGCCG CCAACAGCAA CGGCCTCAAT GCCGCCCGTC TCATGCTAGG CTTGGCTTCA
 6 GCTAATTGAT GCCCACGCTG CCCCCTCTAA TTAATAAATA AATAAATACA CCCTACCTCA GCTCTCAGCA CTTAACTCTA TCATTATATG AGGGCTTAAT
 7 GCATGTACTC TTTCTTTCTT CTATCCTACT TAGTTTCAAA TATATGCACA TATATTCAAA ATAAAAAAAA AAAAAAAAAA ACCGAATGGC CATGGGACGT
 8 CGACCTGAGG GTATTATAAC CCGGGCCCTA TATATGGATC CNATTTGCAT GATCATCATG ACAGATCTGC GCGCGATCGA TATCAGCGCT TTAAATTTGC
 9 CATGCTAGCT ATAGTCTAGA GG
```

The Genbank submission for this sequence is from 40 to 783 (see http://www.ncbi.nlm.nih.gov/nucest/34345996) whereas we suggest not submitting this sequence as it is abnormal.

>gi|68086938|RTMNUT1_27_H12.g1_A029 RECA-Type: A2; EST Dir: 5'; Pattern: N,V,3TSS-2,V **D**

```
          10         20         30         40         50         60         70         80         90        100
 0 TGCACGTAGC GCCTCGCTAG CCTTCGTCCT GGTCCGCCCC AAACGTTTCG GCGAGAAGCA GGCCACTTTT CGCCGGCATG GCGGCCGACG CGCTGGGCTA
 1 CGTCTTGCTG GCGTTCGCGA CGCGAGGCTG GATGGCCTTC CCCATTATGA TTCGAATGGC CATGGGACGT CGACCTGAGG TAATTATAAC CCGGGCCCTA
 2 TATATGGATC CAATTGCAAT GATCATCATG ACAGATCTGC GCGCGATCGA TATCAGCGCT TTAAATTTGC GCATGCTAGC TATAGTTCTA GAGGTACCGG
 3 TTGTTAACGT TAGCCGGCTA CGTATACTCC GGAATATTAA TAGGCCTAGG ATGCATATGG CGGCCGCCTG CAGCTGGCGC CATCGATACG CGTACGTCGC
 4 GACCGCGGAC ATGTACAGAG CTCGAGAAGT ACTAGTGGCC ACGTGGGCCG TGCACCTTAA GCTTGGCACT GGCCGTCGTT TTACAACGTC GTGACTGGGA
 5 AAACCCTGGC GTTACCCAAC TTAATCGCCT TGCAGCACAT CCCCCTTTCG CCAGCTGGCG TAATAGCGAA GAGGCCCGCA CCGATCGCCC TTCCCAACAG
 6 TTGCGCATCC TGAATGGCGA ATGGCGCCTG ATGCGGTATT TTCTCCTTAC GCATTCTGTG CGGTATTTCA CACCGCATAC TTCAAAAGCA ACCATAGTAC
 7 GCCCCCTGTA GCGGCGCCAT TAAGCGCGGC GGGTGTGGGG GTTACCCGCA GCGTGACCGC TACACTTGCC AGCGCCCTAC CCCCCCCTCC TTTCGCTTTC
 8 TTCCCTTTCA TTTCTCGCCA CGTTAGCCAG TATTTCTCCC TCAAGCTTCT AAATCGGGGG GTTCCCTTTT AGGGTTCCTC CATTAGTTGC ATTACCGGCC
 9 CCCCCGACCC CCATAAAAAC TTGATTTGCG TTAATAGGTT CCACCTAATG TGGCCCACCC CCCCTAAATA AAACCGGTTT TTCCCCCTTT TGCCGCTTTG
10 GAATTCTCCC TTTCTTTTAA ATATTGGAAC CCTTTGTTTC CAAAATCTGG AACGACACCT CCAAACCCCT TAATACGAGG GTCAATTTTT TTTTGAATTT
11 TCTTACAGGA ATTTTACACC CACTTTCTCA CTTCTTCTGT TATAAAACAT TGTAGCCGTT TTATTACACT ATAAATTTTA ACACCCGATC TTTATACAAA
12 CAATAATTAC ATCGTCTTAC ACACTATTGA GGGTCTCACC CTCCTCTGAA TAATATCTCG CTCACTTGAT CCCGCCCAAC ATATATATAC ATAACTCCAC
13 AAACCATCTA CACTCAATAT AATTCATAGA TCCTCACCCT CAGAAGAGAT GACTTAGCG
```

The Genbank submission for this sequence is from 30 to 153 (see http://www.ncbi.nlm.nih.gov/nucest/68086938) whereas we suggest not submitting this sequence as it is abnormal.

>gi|66742861|STRS1_37_H01.b1_A034 RECA-Type: A2; EST Dir: 3'; Pattern: 5TNS-2,V,N,3TNS **E**

```
          10         20         30         40         50         60         70         80         90        100
 0 TACAATCTGC CAGTGGCCCT AGTCTTCTCG AGCTCTGTAC ATGTCCGCGG TCGCGACGTA CGCGTATCGA TGGCGCCAGC TGCAGGCGGC CGCCATATGC
 1 ATCCTAGGCC TATTAATATT CCGGAGTATA CGTAGCCGGC TAACGTTAAC AACCGGTACC TCTAGAACTA TAGCTAGCAT GCGCAAATTT AAAGCGCTGA
 2 TATCGATCGC GCGCAGATCT GTCATGATGA TCATTGCAAT TGGATCCATA TATAGGGCCC GGGTTATAAT TACCTCAGGT CGACGTCCCA TGGCCATTCG
 3 CGCAACTGAA CCTGTCGCAT AATTCCCTGT CAGGGATGAT TCCAGTTGGG GGCTTGCTTC AAAAGTTTCC AATATCATCA TACTCTGATA ACAGCGGACT
 4 CTGTGGAGAT CCTCTTCCTC ACTGCTGATC ATATAATAGA TCCTCTTCCT GAATCCATGC GGGGTTAGTG GCGTCTTTTC GTCTTTATGT TTTCTGGTTT
 5 TGCTTTTGGA CTTTCAATAA TTGAGGAGCC TAGTCTCATC TGCTCTCCCA ATCTTAAAAT CTGACATACC AAGCCTGCAC CTATACTAAG GCTTGTATCT
 6 GGTCCATATG GAATAAGTCG TACCAAGCCT GCACCTATAC TAAGGCTTGT ATCTGGTCCA TATGGAATAA GTCGGATTTG TAATAATTTC TCCTCGTGCC
 7 GAATTCGTAA TCATGTCATA GCTGTTTCC TGTGTGAAAT TGTTATCCGC TCACAATTCC ACACAACATA CGAGCCGGAA GCATAAAGTG TAAAGCCTGG
 8 GGTGCCTAAT GAGTGAAGCT AACTCACATT AATTGCGTTG CGCTCACGGC CCGCTTTCCA GTCGGGAACC CTGTCTTGCC AGCTGCATTA ATGAATCGGT
 9 CAACGCCCGG GGAGAAGCGG TTTGCCATTT GGGCGCTCTT TCGCTTCCAT GCTCACGGAA TCACTGCCCT CGGTCTTCCG GCTTCCGCGA GAGGGTATCA
10 GCTCACTCAA AGGGGGGGAA ATCCGGTTAC CCCCTGAATC AGGGGGATAA CCCAGGAAAA AAATGTGTGA GCAAAAGGGC CTCCAAAGGC CCGGAAACCT
11 TAAAAAGGGG GGGTTTCTGG CGGTTTTCCA ATGGGCCCCC CCCACTTGAA AGTCTTAAAA AATTGAACCT TTGTTTAAAG GGGCGAACCC CACCGGGATT
12 TAAAAGATAC GGGGTTTTCC CCGGAAGACA CTCTAGGCGC TTCTTGTTCC ACCCTGCGCT AACGCGAACA TTAGCGCCTT CTTCCATGTG GGAACGGGGG
13 TTTTTCATAA TTCTGGTATA ATTAGACCTG CGGTGGCAGG A
```

The Genbank submission for this sequence is from 300 to 691 (see http://www.ncbi.nlm.nih.gov/nucest/66742861, a reverse complimentary sequence had been submitted) whereas we suggest not submitting this sequence as it is abnormal.

>gi|21689178|NXRV076_A06_F RECA-Type: A3; EST Dir: 5'; Pattern: 5TSS,3TNS-1,V,3TSS-2 **F**

```
          10         20         30         40         50         60         70         80         90        100
 0 CAAAAACTGG AACTCCACGC GGTGGCGGCC GCTCTAAACT AGTGGATCCC CCGGGCTGCA GGAATTCGGC ACGAGGCCTC GTGCCGATTC GATATCAAGC
 1 TTATCGATAC CGTCGACCTC GAGGGGGGGC CTGGTACCCA ATTCTCCCTA TATTGACTCG TATTACAGAT TCACTGCCCG TCGTTTTACA ACATCATGAC
 2 TGGCAAAACC CTGACTTTAC CCAAGTTAAT GCTCTTGCAC CACATCCCCA TTTCGCCAAT TGTCTTAATA ATTAACAGGC CCACACCATT CTCCTTTACA
 3 AACAGTTGCC CATGCTGAAT GTCGAATGTC TAATAGTAAG CGTTCATATT CCGATACAAT TCGATTGAAA TATTTGTTAT ACCAATTCAT TATTTAACCA
 4 ATTAGCCGAA TTCGTCAAAA TCCTTTATAC ATCTAAAAAA TAACACCTAG ATAGGGTTTA CTGTTGTTTC ATATTGTTAC AACATTCCAT TCTCATACAT
 5 CGTCCGCCTG TATCCCCCAT TGGCCACATT ACACGTTTTC ATGTCCCATC TATATATTCT CCCCAACCCC CCCCTTTTTC CATATCCCCT TTCCAACCCC
 6 CCCATCATAA CACATTTATT TTCCCCCCCA CCCCAC
```

The Genbank submission for this sequence is from 157 to 432, which is part of vector sequence (see http://www.ncbi.nlm.nih.gov/nucest/21689178) whereas we suggest not submitting this sequence as it is abnormal.

>gi|18434026|NXRV_013_E07_F RECA-Type: B1; EST Dir: 5’; Pattern: 5TSS-2,V,5TNS-1,N

G

```
           10         20         30         40         50         60         70         80         90        100
 0 NTAGAGTATG GGGGCCCACG CGGTGGCGGC GCTCTAGAAC TAGTGGATCC CCCGGGCTGC AGGAATTCGA TATCAAGCTT ATCGATACCG TCGACCTCGA
 1 GTTTTTTTTT TTTTTTTTTT GTAGAAATTC GTGAAGTTTT TCTCTGGTAA ATTGCAGTTT TCCAAATACA GAGGACAAAA TTCGATGCCC CCAAATCGAA
 2 ATGGCGAAGA AAACATAATC AACACTCTGA TACAAACTTT GCATATGCTG TACTACTTTA TTATCAATAC ACTAAACAAA ACAAATGGCT TCAACCCCAT
 3 ATATTAGAGC GTTCTCCTTA TGATCAGAAA GCCTTTATGC TTGCAATAGA CAAGCAGAAA CAAGGATTGT TTTCAATAGA CACGCCGGCA AAGGGTGACG
 4 CCGTCACCGA CGGGGATTTG GCTGATCTCA AGGCGGGGAT CGACAGCAAG GGCCTTGTTT AGCTCCATCA CGAAATCCCT GTAATATCTC ACATATTTCC
 5 TCAGGGGAGC ATCGGGGGGA GCCACCACAG ATCCGTTCCA CAGGGTGTTG TCAAATGCAA TCAGACCTCC AACCTTCACC AGATCGATCA GACGCTTGTG
 6 GGTAGTTTAG ATAGTTGTTC TTGTCCCGCA CCCACGAACA CAAAGTCAAA CGATTCATGC ATGTCCTCAT TCCTAAGCAG TTCGTCCAAA ACTGGCAGAG
 7 CAGGGCCCCC TCTGAAATCA ATCTTGTGGG CAAATCCGGC TTTCTCAATA AATAAGCAAT CCGATAGCAA AGTTCTCCCC GGTTGAAGGT CCAGGGCTAA
 8 AAACCTTTTC ATCACCGGGC AAATGCAAGG GCTGGTCCTG AAGAACCCAG TAACCATTTT TACACCCCAA ACCGCCAAGG GGGGTTCTTG GGCGTTTAAA
 9 TGACCCTCAA GCAAGAAGGC CCAAAAAATT TGACCCCCCA CCCGGCAGAA AATTATTCAT GAAGGTCTCA AGGGAATGCT TGGGGAAATC AATTCCTCGG
10 GGGCCTCCTT CCATGTGGGC CAAGGCCCAA GAAAGGGTAC CAACACCCGT TTCTCAATAA TAAAACGGAA TAAAGGGGCA GTCTCCCGGC GAATAAAAAC
11 TTTTTGTGTT CTCAATTTCC TTAAATGGCG GGCGCCCGTG CCCAAATTCT GGAACGAAAG GGAACTAATT TCTTTCGGCA GTATTTTTTT GGGACAACCC
12 TTGGGGGCAT TTCCTCACGG GCTTTGTACA AAGGAGGGCC GCCG
```

The Genbank submission for this sequence is from 122 to 351 (see http://www.ncbi.nlm.nih.gov/nucest/18434026) whereas the cDNA in this sequence is not sense but non-sense and we suggest not submitting this sequence as it is abnormal.

>gi|34488588|RTDS1_2_A09.b1_A015 RECA-Type: B1; EST Dir: 3’; Pattern: N,3TSS-1,V

H

```
           10         20         30         40         50         60         70         80         90        100
 0 CAGCTTAGGT GACGGCCCAC GTGGCCACTA GTACTTCGTG GTAGTGGACG CTTCGGTGGA GGCGGCGACA GCTATGGCTA CGATGCCAAT GGACAGGACC
 1 GCCCTCCCCG TCAACAGTTC GAAGACACCA ATACCTTCAC AGGCACAGAC AACTGGGATA CCCCTGAAGT GTCAGTTGTT GATGAAGCTA AAAATGTGGA
 2 GCCTGAACAG AAGAAACCAG AAGAAGAGGC TACACCAGGG GTTACCTCTG AAAATAAAGA TAACAAAGAA GAGGAGGACA ATGAAATGAC TCTTGATGAG
 3 TATGAGAAAT TATTGAATGA GAAAAGAAAA ACATTGGAAG CTTTAAAAAA TGCGGAAAGA AAGGTTATTC TGGACAGAGA TTTTGAGAAA ATGCAGCTTG
 4 TTGATAAGAA AAATGATGGC ATTTTCATCA AACTGAACTC AGAAAAGGAG AGACAAAGAA AGAAGGAGAC CCTTGAAAAA GAGGAAAAAG CCCGTAAGTC
 5 TGTGAGCATA AATGAGTTCT TGAAACCAGC TGACGGTGAG AGATATTTTA CCCCATCTGG CACTCGTGGG CGTGGCCGTG GCCGTGGACG AGGCCGTGGG
 6 GACGGTGTTA GCACTAGAGG AGGTTTTGGA GGGAGGTACA GTGATGCTGA TCAGGTTGCT GCTCCTTGCA TTGAGGATCC TGGACAGTTT CCAGTACTGG
 7 GTGGGAAGTA ATGTTATCAG CATCACTAAG TGCGTTTTTT GTTAGCTAAA TTANGTTTGT GTTCTGTAAG TCTATCACAA TTACAAAGGC AAACTGTCTT
 8 CCGTGAACAA AGACTCATTA GTGGAAACNA TTTTATTNAT ATTTATAGAA GTTTATGATA TATTAATATT GCAAAAANNA AAAAAAACT CGAGCTCTGT
 9 ACATGTCCGC NGTCGCGACG TACGCGTATC GATGGCGCCA GCTGCAGGCG GCCGCCATAT GCATCN
```

The Genbank submission for this sequence is from 38 to 804 (see http://www.ncbi.nlm.nih.gov/nucest/34488588, a reverse complimentary sequence had been submitted) whereas the cDNA in this sequence is not non-sense but sense and we suggest not submitting this sequence as it is abnormal.

>gi|67195335|RTFEPL1_26_F12.g1_A029 RECA-Type: B2; EST Dir: 5’; Pattern: V,N,3TSS

I

```
           10         20         30         40         50         60         70         80         90        100
 0 NNCCCGTGTC GATGAGAGTC NACTGAGGTA TTATAACCCG GGCCCTATAT ATGGATCCAA TTGCAATGAT CATCATGACA GATCTGCGCG CGATCGATAT
 1 CAGCGCTTTA AATTTGCGCA TGCTAGCTAT AGTTCTAGAG GTACCGGTTG TTAACGTTAG CCGGCTACGT ATACTCCGGA ATATTAATAG GCCTAGGATG
 2 CATATGGCGG CCGCCTGCAG CTGGCGCCAT CGATACGCGT ACGTCGCGAC CGCGGACATG TACAGAGCTA AAAACCTCAA TTTATTATCT ATACTAATAA
 3 CTTTAAAAGA AAAACCAGAT ACAAAACCTC AAATTCTGTC ATCTCCAAAT TATAAACTAA GGCAATGCGC ATTGATTGGA TCACACAGAT ATCTTTGATC
 4 CCCTCTTGCA ACACAAACAC AAACACATTA CTAATCAAAG TCCCCACAAC ACGCACATAC GTTTATTATC ATCCACGCTC CTAAGCAAAG GCCTGAAGGA
 5 CCAAAAACAG ATACAGATTC ACATGTTTAC AACATCTTTA CTGATGGTGC TTCCTCCGCC TCTTCCCCGC CATTCTTCTG GTTCTAGTGT CCAGGCAGCT
 6 TCTCTTTGAT TTTATCGAGC AGACCCGCCT TCTTCTCCTC TTCACCCCCG TGCTGTTCGG AAGAGTGCTT TCCGTCACCG GAGAGCTTTT CTTGGTGTCC
 7 CGGAATCTTC TCTTTGATTT TATTCAGCAG ACCCGTCTTC TCCTCTTTAC CCCCACGCTC ATCTGAAGAA TGCGTTCCGC CGCCGGGTAG CTTTTCCTTG
 8 ATCTTGTCCT TCAACCCTTT CTTCTTCCTC TCGCCTTCTT TCTTCTTTTC GCCTTTCTCT TCTTCATCGC TCGACCGCAA TTAATTATAT ATATATATAT
 9 ATAATATACC ATAACAAAGG GGAAAAAAAA CTCGAGAAGT TCTTATTGGC CCCGTGGGGC CGTGGACCCT TAAGCTTGGG CATGGGCCGT CGTTTTTAAA
10 ACGTCCGTGA CTGGGAAAAA CCCTGGCGTT TCCCCACTTA AATTGCCCTT GAAACAAATC TCCCTTTTCG CAAGTGGGGC GAAAATACGA AAAGGGCCCC
11 ACCCGATTTG CCCTTTCACA ACATTTTGCC CAACCTGAAA GGGAAAAGGG GCGCCGAAAG CGGTTTTTTT CCTCCTTACC AAACTGTGTG GGGTTTTCCA
12 CCCGGCAACA CTTAAAACCA CCCTATTTAT CCCCTCCTGT AGGGGCCCTT AAGCCCGGGG GGTGTGTGGG GGTTACCGCA ATGGAAACAC ATAATATTGT
13 CGGGTCCGAT AGCCCCCCTC CATAGTGTTA TATCTCTTTC TTTTG
```

The Genbank submission for this sequence is from 270 to 839 (see http://www.ncbi.nlm.nih.gov/nucest/67195335) whereas we suggest not submitting this sequence as it is abnormal.

>gi|67195189|RTFEPL1_26_F12.b1_A029 RECA-Type: B2; EST Dir: 3’; Pattern: 5TNS,N,V,3TNS-2 **J**

```
          10         20         30         40         50         60         70         80         90        100
0  TTTAAGCAGG CGCCAGTGGC CCTAGTCTTC TCGAGTTTTT TTTTCCCTTT GTTATGTATA TTATATATAT ATATATATAA TTAATTGCAG TCGAGCGATG
1  AAGAAGAGAA AGGCGAAAAG AAGAAAGAAG GCGAGAGGAA GAAGAAAGGG TTGAAGGACA AGATCAAGGA AAAGCTACCC GGCGGCGGAA CGCATTCTTC
2  AGATGAGCGT GGGGGTAAAG AGGAGAAGAC GGGTCTGCTG AATAAAATCA AAGAGAAGAT TCCGGGACAC CAAGAAAAGC TCTCCGGTGA CGGAAAGCAC
3  TCTTCCGAAC AGCACGGGGG TGAAGAGGAG AAGAAGGCGG GTCTGCTCGA TAAAATCAAA GAGAAGCTGC CTGGACACTA GAACCAGAAG AATGGCGGGG
4  AAGAGGCGGA GGAAGCACCA TCAGTAAAGA TGTTGTAAAC ATGTGAATCT GTATCTGTTT TTGGTCCTTC AGGCCTTTGC TTAGGAGCGT GGATGATAAT
5  AAACGTATGT GCGTGTTGTG GGGACTTTGA TTAGTAATGT GTTTGTGTTT GTGTTGCAAG AGGGGATCAA AGATATCTGT GTGATCCAAT CAATGCGCAT
6  TGCCTTAGTT TATAATTTGG AGATGACAGA ATTTGAGGTT TTGTATCTGG TTTTTCTTTT AAAGTTATTA GTATAGATAA TAAATTGAGG TTTTTAGCTC
7  TGTACATGTC CGCGGTCGCG ACGTACGCGT ATCGATGGCG CCAGCTGCAG GCGGCCGCCA TATGCATCCT AGGCCTATTA ATATTCCGGA GTATACGTAA
8  CCGGCTAACG TTAACAACCG GTACCTCTAG AACTATAGCT AGCATGCGCA AATTTAAAGC GCTGATATCG ATCGCGCGCA GATCTGTCAT GATGATCATT
9  TGCATTGGAT TCTTATATAG GGCCCGGGGT TATAATTACC CCAGGTCGAC GTCCCATGGC CCTTCGAATT GCTAATCAGG TCATACCTGT TTCCCGGGTG
10 AAAATGTTAT CCGCTCCAAA TTCCCCCCAA CTTAACAGCC GGAAGCCATA ATTGTTAAGC CCGGGGGGCC TAAGGAGTGG GCCTAACTCC ATTAATTGCG
11 TTGCCCCCAC TGGCCGCTTT TCCGTCGGGG AAACCTGTCT GGGCAGCCTA CTTTAATGAA TCCGCCCAAC CCCGGGGGAA AAGGGGGGTT TGCGTATTTG
12 GGGGCTTTTC CGGTTTCCTC CCTACAGAAT CTCTTGGCCT CTGGTTTTTT CGGGCGCGGC GAGGAGTATT CTACTTCCTC TAAAGGGGGT ATAAAGGGTT
13 ATTCC
```

The Genbank submission for this sequence is from 28 to 695, including the restriction enzyme site (see http://www.ncbi.nlm.nih.gov/nucest/67195189, a reverse complimentary sequence had been submitted) whereas we suggest not submitting this sequence as it is abnormal.

>gi|NULL|NXCI_011_D03_F RECA-Type: C; EST Dir: 5’; Pattern: 5TSS-2,V,3TSS-2 **K**

```
          10         20         30         40         50         60         70         80         90        100
0  TGATATGAGA CATGCTGGAG CTCCACCGCG GTGGCGGCCG CTCTAGAACT AGTGGATCCC CCGGGCTGCA GGAATTCGAT ATCAAGCTTA TCGATACCGT
1  CGACCTCGAG GGGGGGCCCG GTACCCAATT CGCCCTATAG TGAGTCGTAT TACAATTCAC TGGCCGTCGT TTTACAACGT CGTGACTGGG AAAACCCTGG
2  CGTTACCCAA CTTAATCCCC TTGCAGCACA TCCCCCTTTC GCCAGCTGGG GTAATAGCGA AAAGGCCCCG ACCGATCCGC CCTTTCCAAC AGTTGCGCAC
3  CCTGAATGGC GAATGGCAAA TTGGAAGCGT TAATATTTTG TTAAAATTCG CGTTAAATTT TTGGTAAATC AACTCATTTT TTAACCAATA AGGGCGAAAT
4  TCGGAAAATT CCTTATTAAT CAAAAGAATT GACCGAGATA GGGTGGAAG GTCTGTTCAA TTTGGAACAA TAGATCCACT ATTAAAGAAA CGTGGAACTT
5  CTAATGTCAA AAGGGCGGAA AAATCCGCAT TATCATAGGC TGATAGCGGC CATCCTTACC TATGAACCCA CCTTATCTCC TCAAATTAGC AATGTGTTGT
6  TTGCGGACGT CTTAGTAGCT TTCCTAAGCT TACGCCTACG TCCAGATTAT CTCACGATGA CCGCTTCGCT AACGCTAGTG TCATCATTTC TGTACTGTCT
7  ATAGTACAAT GCTCTGTCTA TCGCGTACGG CTCACGGTCT TTCGTTCATC GTTCACCCGC TCCTCTCTAC GTCC
```

There is no Genbank submission for this sequence. Actually there is no cDNA fragment detected in this sequence.

>gi|NULL|NXCI_029_D07_F RECA-Type: C; EST Dir: 5’; Pattern: 5TSS-2,V,3TSS-2 **L**

```
          10         20         30         40         50         60         70         80         90        100
0  TGTTGGACAA GCTGGAGCTC CACCGCGGTG GCGGCCGCTC TAGAACTAGT GGATCCCCCG GGCTGCAGGA ATTCGATATC AAGCTTATCG ATACCGTCGA
1  CCTCGAGGGG GGGCCCGGTA CCCAATTCGC CCTATAGTGA GTCGTATTAC AATTCACTGG CCGTCGTTTT ACAACGTCGT GACTGGGAAA ACCCTGGCGT
2  TACCCAACTT AATCGCCTTG CAGCACATCC CCCTTTCGCC AGCTGGCGTA ATAGCGAAGA GGCCCGCACC GATCGCCCTT CCCAACAGTT GCGCAGCCTG
3  AATGGCGAAT GGCAAATTGT AAGCGTTAAT ATTTTGTTAA AATTCGCGTT AAATTTTTGT TAAATCAGAT CATTTTTTTA CCAATAGGCC CGAAATCGCG
4  CAAAATTCCT TATAAATCCA AAATAATTTG ACCCGATATT ACGGGTGGCG CCCTTTTTTC CCACTTCTTC ACTATTATAC GCCCTCTTTT TAACGTATGT
5  GGCTCTCCAT TTATCCCAGT CGTTACGGAA TCATGCTATC TACTCTTCCT CCTTTCCCAC ACTCACGTTC GTACCTCTTT TTGTCTTTCC TTCCTAACGT
6  TTACTACTGT CTCATACCTC CTTCACCAGA TCACCCTATA TGTAATGACT CCCTATACGC TCTCATCTAA TTCCATACCC ATTTCTCTAC GTATGCTTAC
7  TATTCCATAA ATCCATCGTG TCTCACCTCA TCCTCACTAT GTATTTCTTC CTGTTCGTTG TTCGCTCC
```

There is no Genbank submission for this sequence. Actually there in no cDNA fragment detected in this sequence.

M

>gi|37563947|RTCNT1_24_B05.g1_A029 RECA-Type: D; EST Dir: 5'; Pattern: N,3TNS-1,V,5TNS-1,N

```
           10         20         30         40         50         60         70         80         90        100
0  TTTTTTCAAA AGACGGTCAC AAGCGAATAA ATAGTGAAAT GTTATAAGGC AACATTTAGG GCAGAAGAGA TGGACAACAG TCATGTTAAA AAGAAGATTA
1  ATTAAAAACT ACATTACAAC ACGTAGTATT GGTTAATTAC CCATCTAATT AACAGGTCGG CGGCTTAGGC GCGTTGGTAA GTACGCTTGC TGGTGACGTC
2  CTCACACTTG CAAACAAGGA TCAACTTGTC GCCTTCAACG TAACGCTCAA AAGAGCTTTC TTTGTCGCCA GCTTTGATTG CTTTTTGAGT TTGAAGAAGA
3  GACTCCAGCC TCGTGCCGAA TTCGAATGGC CATGGGACGT CGACCTGAGG TAATTATAAC CCGGGCCCTA TATATGGATC CAATTGCAAT GATCATCATG
4  ACAGATCTGC GCGCGATCGA TATCAGCGCT TTAAATTTGC GCATGCTAGC TATAGTTCTA GAGGTACCGG TTGTTAACGT TAGCCGGCTA CGTATACTCC
5  GGAATATTAA TAGGCCTAGG ATGCATATGG CGGCCGCCTG CAGCTGGCGC CATCGATACG CGTACGTCGC GACCGCGGAC ATGTACAGAG CTCGAGTTTT
6  TTTTTTTTTT TTTTATAAAA AACACACTGA TGTATCAGTA GCATTACTCA AATTTCTGTA TTACACACTT TTTCAACATA TTACTCATAT TTCTGTATTA
7  ACCAAACTTT TTTCAACATT ATAAAAAATC ACACTGATGG TTTCCGTAGT ATTACACATA TTTCCTGCTG GCCTTCTGGG AAACTTAATA CCCAATCCAA
8  CATATTTTGA ACTATTTCCA TTTTCAATTT AGGTAAAAGG GAATTAATGG GATGCCTCCC CCAAATGGTA AAAAACACN
```

The Genbank submission for this sequence is from 10 to 323 (see http://www.ncbi.nlm.nih.gov/nucest/37563947) whereas there are two cDNA non-sense (not sense) strands in this sequence and we suggest not submitting this sequence as it is abnormal.

N

>gi|57882901|COLD1_26_G12.b1_A029 RECA-Type: E; EST Dir: 3'; Pattern: 5TNS-2,V,N

```
           10         20         30         40         50         60         70         80         90        100
0  NNAACAGCAT GCAACAGNGG CCCTANACTT CTCGAGCTCT GTACATGTCC GCGGTCGCGA CGTACGCGTA TCGATGGCGC CAGCTGCAGG CGGCCGCCAT
1  ATGCATTTTT TTGAACGAAA TGTCGAGATT TTTATACCAA CTGTAAGTGT TCGAGCGATC TCCAGTTCAA GACATGTCAT GACCATGAAC AAAGGATTAA
2  TAATTGTTCT GCAAAGCTCC AGTGTAATTT TTCATAATAC AGCAGTTAAT CCATTTCATC ATCAGGATGA ATTGTAATTA ATCCTCAATT ATTCTATGGC
3  ACAATATAAA AAAAATTAGA ATATTGAACT GATGCAATGG CACCTAGCAT CTAAAACTTG GATACCAAAG AAACTAAGGT CGTTTCGAGG GCCAAAGAAA
4  TAGTAGCACC AGTTTGGAAT ATAAAATAAG AGCCTGCTTC AAGCAACAGG AGGCCTTAGC ACGTGATCCT GGGAAGTATC AACCACAGCT GGAGGCATCC
5  ATTGCCACAT TGCCATTCCA GGATATCCTG GAACAGGCAT AGTTTTGTTT GCTGCTGCTT GACTTTGGGC TGCAAAAGCA GCAGCCGCAT GAAATGCTGC
6  AGGATGCGGC ACAAAGCCTG TGGGCAAAGC CATTGCTTTG ACTTGTTGCT CCAATTTTTC CTTTTCTGCT TTCATTCTCA GCTTTTCATC ACGAAGTTCA
7  TTTTTCTCTG CCTTCAGATC TTTAATGGCT TCCTGCAGTC GTTCATTCTC AGCTTTCAGG TTCTGCGCCT CAGTTCGTAG CTGGGTCATA ACACGTGCAG
8  CATCAGACAA AATTGTGGCT TTGTCTGCCG TCTTGGGAGG CCTACCCGGT TCTAACACAG AGCTTAGCTC CATGAACCTA TCATTAAGTC TGTCCCTCCG
9  CATCTTCTCA CGGCAAGCTT TGGACCCAGG GTCACCACTA GATTCTTTCC GTGACCCTCT TCTAGGACAT AATTTATCTT GCTCGTCCAT GTCCTTATTG
10 GGGCCATCAA ATTCTACACT ACCACTTACG TCGGGCTGAT CATTTATGCC CTGACCGCAC AGAAGAATGG ATTGGCCGGC TGCCAACGCC TACTCATCCA
11 AAATGGCTCG TCAATTATAA ACCCTCGGGG CCAAAATTCG AACAGGGCAA ACCGTTACCG GGGGAAATTT TACCGCCTCA ATTTCCAACA AATACGACCC
12 GAAACCAAAG GTAAACCCGG GGGCGCAAAG AGGAACACAC CGTTATTGGT GGGCCCCACC CCCTTTCTTG GGGAAACATT GGGCCCTCCT TAAAATACCC
13 CCCCGGGGAA GAGGTTTAAT AGGGGCCTCC CTTCCAAAAA AACACCGGGG GTGT
```

The Genbank submission for this sequence is from 107 to 932 (see http://www.ncbi.nlm.nih.gov/nucest/57882901; a reverse complimentary sequence had been submitted) whereas we suggest not submitting this sequence as it is abnormal.

O

>gi|66977581|RTCA1_14_E09.g1_A029 RECA-Type: F; EST Dir: 5'; Pattern: V,N

```
           10         20         30         40         50         60         70         80         90        100
0  NNNCCTCCGA TGAGGAGTCN ACTGAGGTAT TATAACCCGG GCCCTATATA TGGATCCAAT TGCAATGATC ATCATGACTT TTGAACGTTG GAATCATTAA
1  ATTTTTATTT CATTTTGCTA CATCAAAACC GGGCCATCCT ATAAGTAAAA CAAAACAGAG TCATATACAT GACGAACAAC TTGACGAGGC ATTATTCCTA
2  AGTAGAATCT ATTTAGGGAG TTTCCTTGTA ATAACCATTG TCGGCAACAT ATTCTGCGTT GCCCATGCCG GAGCCCGCTT CGTAGCCATT ACGGTTACCG
3  GAACCGGTGA CATAGCCACC AACATAACCT GAACGGTAGG CGCCATTCCT TTCCGATCGT CCGCCGTAGC CTGAGGCGTC TTGGTCACCA CCTTGCCCAT
4  AGCCACCATT AGAGCCACGC CAGTCGGAGT CGGATCCAGA ACCAGATCCC GAACCATAAA CTGCGCCGCG GTAGCTAGAG TCCGCGCCAT AACCAGCGCC
5  GTTACCAAAA CTCTCTTTAT ATGCGGCACC GGCGACGTTC CCGGCATTGT TTTGTGAATA GTACTCACCA GTTCTACCAG AACCGGAACG ATAGCCGCTC
6  CCGGCTTTTC CATAGCCGGC TTTACCGCCA AATCACCGTG GTAAGCCTCG CTGTCGCCAT ATCCATATCC ATTACCGTTT CCCTGGGCTC GATAAGAACC
7  GTAAACATTG CCCGAACCTG CTCCGTAAGC TTTATCTACA CCGCTGGTGA CGTAGCCTTC CTCGTACCCG GCGGTATTTG TTCACCATTA AGGGTGTCGT
8  AGTTAGGCCC CTTGTTAAAG AATCTGGTCT TCCTCGCCTC CACGTTACGG ATAAAACAAC TCCAAGCAAG CCCTTTTATG CCCAAGACTT TAGTCAGGGC
9  GGGGAGCCTT TCCGAATAGA ATCTCAATAT GGAGCTCACA GGGACTAGCC TAACTCTAAA GCTCGGGGCA AATTCGGCAA AAGGGCCAAG AAGGGAAAAT
10 TTTCGCAAGT CATTTTCCTT TTTCGGGCGA AACATTCTGG GTTATTCCAA GTCAAAATTC TGCAGTTAGG GCCCCCCGGT CCCCAAGCCG ACGCTTAATT
11 TTTCCCCCTC CCCCAAAAAT TCCCGCCCGG GGAAAACCCC CCCCGTTTTT ACCCCGCGGG GAAAAACCCC CCCGAAATTC CCCCCCCTGT TTTGACAACC
12 CCCACCTGGG AATTCCCGGT GTTCCCCCCA AAATTCCTTC TGCGGAAACT CCCCCGGAAA ACCCCTTTAA GGGCCCCAAA AGACCCCCGA GCTCCGGGTA
13 GGCCCCCTAA TACCGGGGAG GCCCCCTTGT TTCTAAAAAA GAATGAGAGC CCCTGTTTCC CTCCCCCACG AT
```

The Genbank submission for this sequence is from 79 to 667 (see http://www.ncbi.nlm.nih.gov/nucest/66977581) whereas we suggest not submitting this sequence as it is abnormal.

Supplemental Figure 3 (S3)

>gi|57885055|COLD1_44_G01.b1_A029 EST Dir: 3’; Pattern: N,5TNS-5,DBT,DBT,DBT,N

A

```
            10         20         30         40         50         60         70         80         90        100
0  NNNAAGCGAC ACGCCACTGG CCACAGTACT TCTCTCGTGC CGAATTCGGC ACGAGGCTTC GTGCCGAATT CGGCACGAGG CCTCGTGCCG AATTCGGCAC
1  GAGGCTATAT TCTTCTATTC AAGACGCTTG CAGGATTCTT CTTAATTATG TCCTGCACTA TACTTCAAGC TATTTGTTTG ATTCTCAAAA GTGGTCGGAC
2  AGGATCACAA AGAGAAGCCC ATATAGACCT GTTGGCACTT ACATGTTCTG CTCAAGCCGT GGCGCAGCTT GTATTGGGAA TTCTGACACC GTTTTGAAGA
3  TGCTTCATTT GACGATGCAG AGCCATGAAA AGCCCTGGGA TATTGCACTG GACGACTGTT TTTCAGAGCA TATAGTGTAT GCTTCGGTTT TGAAACATGT
4  CATAGAAAAC TCCAAAAATG GGAGAAATTT TGCATTTAAT CCTGATTCAG AATTTTCTTA CGACAGGGGA ATTTCACTGC ACTTAGAAGC TACTGGTGTT
5  GGAGCTCACA ATGATCATGC ACAGCTTGCC CTACGAAAAG CTGGAGAAAC TGAAGAAAGG TATAACACAC ATGTTGACGA GCTAGCTATT GAATTAAGCC
6  TAAAACAAAG TAGCATGGCA GAGCTTGAAT GGTATAAAGA GAGTTGTGAG AAGGATGGTG ACATCGGATA TTATGATGCC TTCAAGAACC ATAACATGAA
7  GAGCGATATC GAAGCGAATT TGCGTAGGGT AAAGCTTGCA GAGTTTTGGG ATGAGATTAT TGAACAGTGT GAAGGTCATG ATTTGCCAAG TGATTTCGAA
8  TCCCACAATA AGTGGATAAA TGCTGGCAAT ATATTAACAA AACTGGCTGA GCCTCTGGAT ATACACATTA TTATCCCAAT ACCCCCTTGG GTACTACCTT
9  CAAATGGAAA CCCAAACTCC ATGAGGTTCT TTCAGAATGG ATGGAACCGA AAGAAAAAAC TCCCATTTAA GAATTCAAAA AATAGCCTAA AGCCTGGCTC
10 CCTTTATGAA GAACCGTGCT TTTGGGCTAT TTAGAAGAAG CTTGTAATAA CAGTGAAATC TTAATAAGGC CACCACAAAA CCTACAATAC TTTTAATTTC
11 TAAAAATTTG ACAATGGGGG AAAAGCTCCT ACCTTTTTAC AATTTTCTTG GAGGATAGGG TTTTTATTGC GTGGGAAAAC AGAAAAGTCA AAAAATTATT
12 GGCAAAGGGG ACTCCCTTTA ATCCAAATGG AAAACCTATT GGAAACACCT GCGCCCTCCA AATTATAATT TTCTCAACAA TTTTCCGTTG GGGGAAATTT
13 TATGAAAAAT CAATGTTTAT GTTTAAATTG GGCGAGATAT TCTATATTTT GGAAATTTCT ACTAATGACA TATTCTTTTG
```

The Genbank submission for this sequence is from 40 to 713, with DBTs that should have been trimmed off (see http://www.ncbi.nlm.nih.gov/nucest/57885055, a reverse complimentary sequence had been submitted). We suggest not submitting this sequence as it is abnormal.

>gi|48932499|FLD1_32_F06.b1_A029 EST Dir: 3’; Pattern: N,5TNS,N,DBT,3TNS,V

B

```
            10         20         30         40         50         60         70         80         90        100
0  NGGGGTCGGC TTCGGCCCGC GGCCACTAAT ACTTCATCGA GTTTTTTTTT TTTTTTTTTT TGGTTCTAAA AAGAAAAGAA AATATATTTA CTTTCACTGG
1  TGAAAATATA CATAGTGCAT AGAGGAAATA ATATTACAGT CAAATATTAG AACTGGCAAT ATTGAAATGC CTTATTTTAT ATCTTCCCAA TACTTGGGTG
2  CTTGGAGAAA TTCATTGTGA AAATTGAGCA AACTATTGCT GTGCAGACAC AAGCAAGGAC AAATACTCTG CAATGGCCTT GCCTTCTTCT TCAACTTCAA
3  TGTCATAGTC TGCTAGTGGA TCTCCAGTCA AGTTATTTAC AATATCTTCT TCCATCTCCT GATCTCGCAC TTCCACGGGT GCACTATCAT GTTCATTGTG
4  ATTTTCTGTG ACATCTTGCA TCTCGTCTAC AGTATCAGTA GCATTTTGGT GTCCTCCTTC CTCAAAGGCT GCAGAACCTA TCTCTGCATG GCCCTCACTA
5  GCAGCACCCG AACCTGCACC ATTTTGGAGT TCTCTAAGAA GGAAGTCTAC TGCCTCTTCC TTTGTACCTT TTCCTCTTTC AAAGCCCATC GCTATCATAT
6  CTGCAATATC TTCCTGCCGG AGAGATTTTT TCCTCTTTCT TCGCCTCGAC TCAAGACTAC CCTGTAGCCT CGTGCCGAAT TCGGCACGAG GCCTCGTGCC
7  GAATTCGTAA TCATGTCATA GCTGTTTCCT GTGTGAAATT GTTATCCGCT CACAATTCCA CACAACATAC GAGCCGGAAG CATAAAGTGT AAAGCCTGGG
8  GTGCCTAATG AGTGAGCTAA CTCACATTAA TTGCGTTGCG CTCACTGCCC GCTTTCCAGT CGGGAAACCT GTCGTGCCAG CTGCATTAAT GAATCGGCCA
9  ACGCGCGGGG AGAAGGCGTT TGCGTATTGG GCGCTCTTCC GCTTTCTCGC TCACTGACTC GCTGCGCTCG GTCGTTTCGC TGCGGCGAGC GGTATCAGCT
10 CACTCAAAGG CGGTAATACN GTTATCAACA GAATCAGGGG ATAACGCNGA AAGAACATGT GAGCAAAAGG CAGCAAAAGT CAGGAACCGT AAAAAGCGCC
11 GTTCTGGCGT TTTCCATAGC TCGCCCCCTG ACAACATCAA AAATTCACTT AAGTCAAGGT GCAAACCGAC GGACTTAAGA ATCAGGGTTT CCCTGGAGCC
12 CTTGGGGTTT CGGTTCCACC GTCCCTACGA AAAGGTCCCT TTTCCTTGGG AAGGGGCTTT CAATCCCCCG GAGATCATTG GGGGGGGTCT CCACGGGGGG
13 GCAACC
```

The Genbank submission for this sequence is from 66 to 691, with DBTs that should have been trimmed off (see http://www.ncbi.nlm.nih.gov/nucest/48932499, a reverse complimentary sequence had been submitted). We suggest not submitting this sequence as it is abnormal.

>gi|48942860|NDL1 11 A06.b1 A029 EST Dir: 3’; Pattern: 5TNS-4,N,3TNS-5,N,3TNS,V

C

```
            10         20         30         40         50         60         70         80         90        100
0  CGCAGCTTAG GTGCCGGCCC ACGTGGCCAC TAGTACTTCT TTTTTTTGTT TTTTTTTTTT TTAAATGCAA GAGTAGGCAA CTCAGATTCA TGCAAAATAA
1  AAATATTTAT TACATAGAGA TTGAGTCATT TGCTATTAAA TGACATGTAT CCCACCCTAT TTGCTATCCT GTGAAAAATC TAACAAAATG GAAAGAAAAT
2  ACGCCATCCA GCACTAGGAT GAGCTTGCTC TCAAATTAAA ATTTCAGAGC AAATCTATGC TAAGATTTGT TTAGCATTTG CAACCACATT GTCAGCAGTG
3  AGGCCAAACT CCTTGTACAA GATTCCAATG GGAGCACTCG CACCAAACCT GTCTATCCCA ACAGCCTTTC CCTTGGATCC TAAGTACCTC TCCCAACCAA
4  AAGTTGAACC AGCTTCCACA CTGACCCTTG CAGCAACAGC TGCCGGAAGC ACACTTTCTT TATATGCTGG AGTTTGTTCC TCGAATAATT CCCAACAGAC
5  AAGGGAAACA ACTCTAACAG CTTTCCCTTC ATTCCTCAAA GTAGATGCAG CTTTCTCAGC AATCTCAAGC TCAGAACCAC TACCCATAAG AATAACATCA
6  GGCTTGTTGC CAGAGGAATT GTCACTGATA ATGTAGCCTC CCTTCTCGAC ACCCTCTATT GAAAACCGGC AAGATGTGGG AGTTTTTGAC GGGAGAGAGC
7  AAGTATTGAT GGCCTCTTTC TATTGAGAAC AGCAACCTTG TAAGCCCCAG CTGTCTCCTT TCCATCAGCT GGACGGAACA TCAAAACATT GGGCATTGCC
8  CCTAAAGCTT GCAAGTGCTC TATTGGGTTG ATGAGTGGGC CCATNNCTCT CGAGACCAAT AGAATCATGG GTCATTACAT ANGATACACA GNCTTTCAGC
9  CTCGTGCCGA AATCGTATCA TGTCAAAACC TGTTTTCTGG GGTGAAATTG TATCCGCTCA CATTCCCACC CACATAGAGC C
```

The Genbank submission for this sequence is the reverse complimentary sequence of region from 63 to 812 (see http://www.ncbi.nlm.nih.gov/nucest/48942860), similar to our clean sequence.
